# Multilayer meta-matching: Translating phenotypic prediction models from multiple datasets to small data

**DOI:** 10.1162/imag_a_00233

**Published:** 2024-07-17

**Authors:** Pansheng Chen, Lijun An, Naren Wulan, Chen Zhang, Shaoshi Zhang, Leon Qi Rong Ooi, Ru Kong, Jianzhong Chen, Jianxiao Wu, Sidhant Chopra, Danilo Bzdok, Simon B. Eickhoff, Avram J. Holmes, B.T. Thomas Yeo

**Affiliations:** Centre for Sleep & Cognition & Centre for Translational Magnetic Resonance Research, Yong Loo Lin School of Medicine, National University of Singapore, Singapore; Department of Electrical and Computer Engineering, National University of Singapore, Singapore; Department of Medicine, Human Potential Translational Research Programme & Institute for Digital Medicine (WisDM), Yong Loo Lin School of Medicine, National University of Singapore, Singapore; N.1 Institute for Health, National University of Singapore, Singapore; Integrative Sciences and Engineering Programme (ISEP), National University of Singapore, Singapore; Institute for Systems Neuroscience, Medical Faculty, Heinrich-Heine University Düsseldorf, Düsseldorf, Germany; Institute of Neuroscience and Medicine, Brain & Behavior (INM-7), Research Center Jülich, Jülich, Germany; Department of Psychology, Yale University, New Haven, CT, United States; Department of Biomedical Engineering, McConnell Brain Imaging Centre (BIC), Montreal Neurological Institute (MNI), Faculty of Medicine, School of Computer Science, McGill University, Montreal QC, Canada; Mila – Quebec Artificial Intelligence Institute, Montreal, QC, Canada; Department of Psychiatry, Brain Health Institute, Rutgers University, Piscataway, NJ, United States; Martinos Center for Biomedical Imaging, Massachusetts General Hospital, Charlestown, MA, United States

**Keywords:** phenotypic prediction, meta-learning, transfer learning, neuroimaging, functional connectivity

## Abstract

Resting-state functional connectivity (RSFC) is widely used to predict phenotypic traits in individuals. Large sample sizes can significantly improve prediction accuracies. However, for studies of certain clinical populations or focused neuroscience inquiries, small-scale datasets often remain a necessity. We have previously proposed a “meta-matching” approach to translate prediction models from large datasets to predict new phenotypes in small datasets. We demonstrated a large improvement over classical kernel ridge regression (KRR) when translating models from a single source dataset (UK Biobank) to the Human Connectome Project Young Adults (HCP-YA) dataset. In the current study, we propose two meta-matching variants (“meta-matching with dataset stacking” and “multilayer meta-matching”) to translate models from multiple source datasets across disparate sample sizes to predict new phenotypes in small target datasets. We evaluate both approaches by translating models trained from five source datasets (with sample sizes ranging from 862 participants to 36,834 participants) to predict phenotypes in the HCP-YA and HCP-Aging datasets. We find that multilayer meta-matching modestly outperforms meta-matching with dataset stacking. Both meta-matching variants perform better than the original “meta-matching with stacking” approach trained only on the UK Biobank. All meta-matching variants outperform classical KRR and transfer learning by a large margin. In fact, KRR is better than classical transfer learning when less than 50 participants are available for finetuning, suggesting the difficulty of classical transfer learning in the very small sample regime. The multilayer meta-matching model is publicly available athttps://github.com/ThomasYeoLab/Meta_matching_models/tree/main/rs-fMRI/v2.0.

## 1 Introduction

There is growing interest in harnessing neuroimaging data to predict non-neuroimaging-related phenotypes, such as fluid intelligence or clinical outcomes, of individual participants (Eickhoff & Langner, 2019;Gabrieli et al., 2015;Varoquaux & Poldrack, 2019;Woo et al., 2017). However, most brain-behavior prediction studies suffer from underpowered samples, typically involving less than a few hundred participants, leading to low reproducibility and inflated performance (Arbabshirani et al., 2017;Bzdok & Meyer-Lindenberg, 2018;Marek et al., 2022;Masouleh et al., 2019;Poldrack et al., 2020). Adequately powered sample sizes can significantly improve prediction accuracy (Chu et al., 2012;Cui & Gong, 2018;He et al., 2020;Schulz et al., 2020), so large-scale datasets, such as the UK Biobank (Miller et al., 2016;Sudlow et al., 2015), are vital for enhancing prediction performance. However, for investigations of certain clinical populations or focused neuroscience inquiries, small-scale datasets often remain the norm.

We have previously proposed a “meta-matching” approach to translate prediction models from large datasets to improve the prediction of new phenotypes in small datasets (He et al., 2022). Meta-matching is grounded in the observation that many phenotypes exhibit inter-correlations, as demonstrated by previous studies identifying a small number of factors linking brain imaging data to various non-brain-imaging traits like cognition, mental health, demographics, and other health attributes (Kebets et al., 2019;Miller et al., 2016;Smith et al., 2015;Xia et al., 2018). As a result, a phenotype X in a smaller-scale study is likely correlated with a phenotype Y present in a larger population dataset. This means that a machine-learning model trained on phenotype Y from the larger dataset might be more effectively translated to predict phenotype X in the smaller study. Meta-matching exploited these inter-phenotype correlations and was thus referred to as “meta-matching.” SeeSection 4for further discussion.

In our previous study (He et al., 2022), we trained a deep neural network (DNN) to predict 67 non-brain-imaging phenotypes from resting-state functional connectivity (RSFC) in the UK Biobank. The DNN was then translated using meta-matching to predict non-brain-imaging phenotypes in the Human Connectome Project Young Adult (HCP-YA) dataset, yielding large improvements over classical KRR without meta-matching. Among the different meta-matching variants, complementing “advanced meta-matching (stacking)” (which we will refer to as “meta-matching with stacking”) performed the best (He et al., 2022). Stacking is a well-known ensemble learning approach (Breiman, 1996;Wolpert, 1992) and has also enjoyed utility in neuroimaging (Liem et al., 2017;Ooi et al., 2022;Rahim et al., 2017).

The original study (He et al., 2022) experimented with only one source dataset (UK Biobank). Using multiple source datasets might lead to better generalization for multiple reasons. First, prediction performance tends to increase with larger sample sizes (Chu et al., 2012;Cui & Gong, 2018;He et al., 2020;Schulz et al., 2020). Second, given acquisition, preprocessing, and demographic differences across datasets, training on multiple source datasets might yield representations that are more generalizable to a new target population (Abraham et al., 2017). Third, different datasets collect overlapping and distinct non-brain-imaging phenotypes. Since meta-matching exploits inter-phenotype correlation, training on more diverse phenotypes might lead to better performance. Here, we investigated the performance of meta-matching models trained from five source datasets—UK Biobank (Miller et al., 2016;Sudlow et al., 2015), Adolescent Brain Cognitive Development (ABCD) study (Volkow et al., 2018), Genomics Superstruct Project (GSP;Holmes et al., 2015), Healthy Brain Network (HBN;Alexander et al., 2017), and the enhanced Nathan Kline Institute-Rockland sample (eNKI-RS;Nooner et al., 2012).

One major challenge is the extreme sample size imbalances across source datasets, for example, the UK Biobank is almost 40 times larger than the HBN dataset. Therefore, there might be diminishing returns from adding smaller source datasets despite an increase in population and phenotypic diversity. A second challenge is that the available phenotypes are different across datasets, so training a single DNN to predict all phenotypes is not straightforward. Here, we considered a naive extension of the original meta-matching with stacking approach by training independent prediction model(s) in each source dataset, and then performed stacking on the outputs of the prediction models in the target dataset. We refer to this extension as “meta-matching with dataset stacking.” Because meta-matching can improve the prediction of smaller datasets, we also proposed an alternative “multilayer meta-matching” approach, which gradually applied meta-matching from large source datasets (e.g., UK Biobank) to smaller source datasets (e.g., GSP, HBN, etc), to generate additional features for a final round of stacking in the target dataset.

We evaluated the proposed approaches in two target datasets—HCP-YA (Van Essen et al., 2013) and HCP-Aging (Harms et al., 2018). We found that both approaches performed better than the original “meta-matching with stacking” approach trained only on the UK Biobank. Given the close relationship between meta-matching and transfer learning, instead of performing stacking on the DNN trained on the UK Biobank (i.e., meta-matching with stacking), we also considered a standard transfer learning baseline (Weiss et al., 2016), in which the DNN was finetuned on the target dataset. Of note, meta-matching with stacking significantly outperformed the transfer learning baseline. In fact, the transfer learning baseline was worse than classical kernel ridge regression when less than 50 participants were available for finetuning, suggesting the difficulty of transfer learning in the very small sample regime. Finally, we found that multilayer meta-matching modestly outperformed meta-matching with dataset stacking.

## 2 Methods

### 2.1 Datasets

As illustrated inFigure 1, we used five source datasets for meta-training: the UK Biobank (Miller et al., 2016;Sudlow et al., 2015), the Adolescent Brain Cognitive Development (ABCD) study (Volkow et al., 2018), the Genomics Superstruct Project (GSP;Holmes et al., 2015), the Healthy Brain Network (HBN;Alexander et al., 2017) project, and the enhanced Nathan Kline Institute-Rockland sample (eNKI-RS;Nooner et al., 2012). The models from the five datasets were then adapted for phenotypic prediction in two meta-test datasets: Human Connectome Project Young Adults (HCP-YA;Van Essen et al., 2013) and HCP-Aging (Harms et al., 2018). All data collection and analysis procedures were approved by the respective Institutional Review Boards (IRBs), including the National University of Singapore IRB for the analysis presented in this paper.

**Fig. 1.**
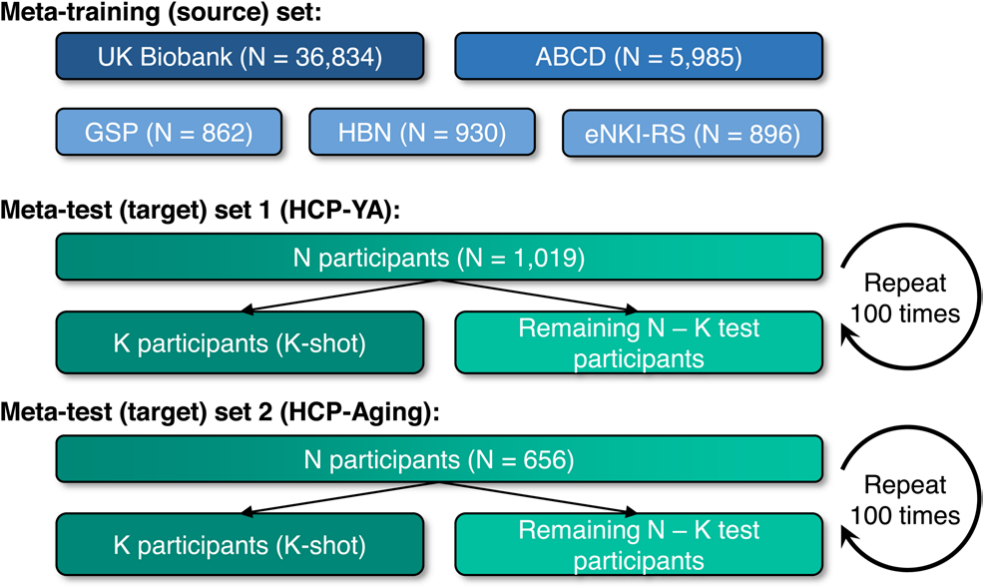
Schematic of meta-training and meta-test sets. Datasets were assigned to meta-training set and meta-test set. Prediction models from the meta-training set were adapted to K participants from each meta-test dataset to predict target phenotypes. The adapted models were evaluated in the remaining N – K participants from the meta-test dataset. This procedure was repeated 100 times for stability. The meta-training set was differentiated into extra-large-scale (UK Biobank; dark blue), large-scale (ABCD; blue), and medium-scale (GSP, HBN, and eNKI-RS; light blue) source datasets.

The summary information of the datasets is listed inTable 1. Detailed information about the non-brain-imaging phenotypes (henceforth referred to as phenotypes) used can be found inTables S2 to S8. The phenotypes covered a broad range of behavioral domains, ranging from cognitive performance, personality measures, lifestyle, and mental health scores. The following subsections describe each dataset and corresponding preprocessing procedures in greater detail.

**Table 1.**
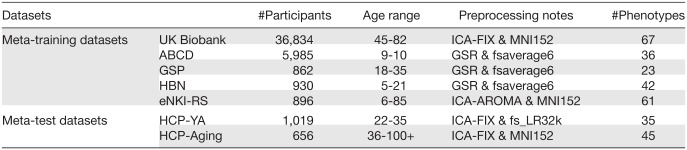
Summary information of datasets used in the current study.

We note that these datasets were opportunistically collated (e.g., by contacting potential collaborators or by downloading preprocessed data provided by the study), so the preprocessing steps varied considerably across datasets. However, we consider the heterogeneous preprocessing as a strength because the heterogeneity might help to improve (and demonstrate) generalization across preprocessing pipelines.

The phenotypes were predicted using 419 × 419 RSFC matrices, consistent with previous studies from our group (Chen et al., 2022;Kong et al., 2021;Li et al., 2022). The 419 × 419 RSFC matrices were computed using 400 cortical (Schaefer et al., 2018) and 19 subcortical parcels (Fischl et al., 2002). We note that the Schaefer parcellation is a group-level parcellation available in fsaverage, MNI, and fsLR space. For each participant, RSFC was computed as the Pearson’s correlations between the average time series of each pair of brain parcels.

#### 2.1.1 UK Biobank

The UK Biobank (UKBB) dataset is a population epidemiology study with 500,000 adults (age 40–69 years) recruited between 2006 and 2010 (Miller et al., 2016;Sudlow et al., 2015). We utilized fMRI data from 36,834 participants and 67 phenotypes (selected from a total of 3,937 phenotypes) from the UK Biobank dataset. The detailed phenotypic selection procedures followed our previous study (He et al., 2022). The sample size is slightly smaller than our previous study (He et al., 2022) because of participants voluntarily withdrawing from the UK Biobank study. More specifically, ICA-FIX pre-processed volumetric rs-fMRI time series in native participant space were downloaded from the UK Biobank (Alfaro-Almagro et al., 2018). The time series were then projected to MNI152 2-mm template space, and averaged within each cortical and each subcortical parcel. Here, the cortical parcels were based on the Schaefer parcellation in MNI152 space, while the subcortical parcels were obtained by FreeSurfer recon-all of the MNI152 template. Pearson’s correlations were used to generate the 419 × 419 RSFC matrices.

#### 2.1.2 ABCD

The adolescent brain cognitive development (ABCD) is a dataset of children (age 9–10 years) and a diverse set of behavioral measures (Volkow et al., 2018). We considered data from 11,875 children from the ABCD 2.0.1 release. We used 36 phenotypes in total, including 16 cognitive measures, 9 personality measures, and 11 mental health measures, consistent with our previous studies (Chen et al., 2022;Ooi et al., 2022).

Details of the fMRI preprocessing can be found in previous studies (Chen et al., 2022;Ooi et al., 2022) but briefly, minimally preprocessed fMRI data (Hagler Jr et al., 2019) were further processed with the following steps: (1) removal of initial frames (number of frames removed depended on the type of scanner;Hagler Jr et al., 2019); (2) alignment with the T1 images using boundary-based registration (BBR;Greve & Fischl, 2009) with FsFast (http://surfer.nmr.mgh.harvard.edu/fswiki/FsFast); (3) respiratory pseudomotion motion filtering was performed by applying a bandstop filter of 0.31–0.43 Hz (Fair et al., 2020); (4) functional runs with BBR costs greater than 0.6 were excluded; and (5) motion correction and outlier detection: framewise displacement (FD;Jenkinson et al., 2002) and voxel-wise differentiated signal variance (DVARS;Power et al., 2012) were computed using fsl_motion_outliers. Volumes with FD > 0.3 mm or DVARS > 50, along with one volume before and two volumes after, were marked as outliers (i.e., censored frames). Uncensored segments of data containing fewer than five contiguous volumes were also censored (Gordon et al., 2016;Kong et al., 2019). BOLD runs with over half of frames censored and runs with max FD > 5 mm were removed; (6) the following nuisance covariates were regressed out of the fMRI time series: a vector of ones and linear trend, global signal, six motion correction parameters, averaged ventricular signal, averaged white matter signal, and their temporal derivatives. Regression coefficients were estimated from the non-censored volumes; (7) interpolation of censored frames with Lomb-Scargle periodogram (Power et al., 2014); (8) band-pass filtering (0.009 Hz ≤ f ≤ 0.08 Hz); (9) projection onto FreeSurfer (Fischl, 2012) fsaverage6 surface space; and (10) smoothing by a 6 mm full-width half-maximum (FWHM) kernel.

We also excluded participants who did not have at least 4 minutes for rs-fMRI and excluded participants without all 36 phenotypes, resulting in 5,985 participants. For each participant, the fMRI time series were averaged within each cortical and subcortical parcel. Here, the cortical parcels were based on the Schaefer parcellation in fsaverage space, while the subcortical parcels (from FreeSurfer recon-all) were projected from the participant’s T1 native volumetric space to the participant’s fMRI native volumetric space. Pearson’s correlations were used to generate the 419 × 419 RSFC matrices.

#### 2.1.3 GSP

The Brain Genomics Superstruct Project (GSP) contains fMRI and multiple behavioral measures from healthy young adults aged 18 to 35 years old (Holmes et al., 2015). We used 23 behavioral phenotypes, including cognitive and personality measures, consistent with our previous study (J. Li et al., 2019).

Details of the fMRI preprocessing can be found in previous studies (J. Li et al., 2019), but briefly, the pipeline comprised the following steps: (1) removal of the first four frames; (2) slice time correction with FSL (Jenkinson et al., 2012;Smith et al., 2004) package; and (3) motion correction and outlier detection: FD and DVARS were estimated using fsl_motion_outliers. Volumes with FD > 0.2 mm or DVARS > 50 were marked as outliers (censored frames). One frame before and two frames after these volumes were flagged as censored frames. Uncensored segments of data lasting fewer than five contiguous volumes were also labeled as censored frames (Gordon et al., 2016). BOLD runs with more than half of the volumes labeled as censored frames were removed; (4) alignment with structural image using boundary-based registration with FsFast (Greve & Fischl, 2009); (5) regress the following nuisance regressors: a vector of ones and linear trend, six motion correction parameters, averaged white matter signal, averaged ventricular signal, mean whole-brain signal, and their temporal derivatives. Regression coefficients were estimated from the non-censored volumes; (6) interpolation of censored frames with Lomb-Scargle periodogram; (7) band-pass filtering (0.009 Hz ≤ f ≤ 0.08 Hz); (8) projection onto the FreeSurfer fsaverage6 surface space; and (9) smoothing with 6 mm FWHM and down-sampling to fsaverage5 surface space.

We also removed participants without full 23 phenotypes, yielding 862 participants. For each participant, the fMRI time series were averaged within each cortical and subcortical parcel. Here, the cortical parcels were based on the Schaefer parcellation in fsaverage space, while the subcortical parcels (from FreeSurfer recon-all) were projected from the participant’s T1 native volumetric space to the participant’s fMRI native volumetric space. Pearson’s correlations were used to generate the 419 × 419 RSFC matrices.

#### 2.1.4 HBN

The Healthy Brain Network (HBN) contains New York area participants (age 5–21 years) with brain imaging, psychiatric, behavioral, cognitive, and lifestyle information (Alexander et al., 2017). We downloaded data from 2,196 participants (HBN release 1–7). We manually selected commonly used cognitive performance scores and behavioral scores with less than 10% of missing values, resulting in 42 phenotypes.

Resting-state fMRI data were pre-processed with the following steps: (1) removal of the first 8 frames; (2) slice time correction; (3) motion correction and outlier detection: frames with FD > 0.3 mm or DVARS > 60 were flagged as censored frames. 1 frame before and 2 frames after these volumes were flagged as censored frames. Uncensored segments of data lasting fewer than five contiguous frames were also labeled as censored frames. BOLD runs with over half of the frames censored and runs with max FD > 5 mm were removed; (4) correcting for spatial distortion caused by susceptibility-induced off-resonance field; (5) alignment with structural image using boundary-based registration; (6) nuisance regression: regressed out a vector of ones and linear trend, global signal, six motion correction parameters, averaged ventricular signal, averaged white matter signal, and their temporal derivatives. Regression coefficients were estimated from the non-censored volumes; (7) band-pass filtering (0.009 Hz ≤ f ≤ 0.08 Hz); (8) interpolation of censored frames with Lomb-Scargle periodogram; (9) projection onto the FreeSurfer fsaverage6 surface space; and (10) smoothing with 2 mm FWHM and down-sampling to fsaverage5 surface space.

We excluded individuals who did not have at least 4 minutes of uncensored rs-fMRI data and removed participants with no relevant phenotypes, resulting in 930 participants. For each participant, the fMRI time series were averaged within each cortical and subcortical parcel. Here, the cortical parcels were based on the Schaefer parcellation in fsaverage space, while the subcortical parcels (from FreeSurfer recon-all) were projected from the participant’s T1 native volumetric space to the participant’s fMRI native volumetric space. Pearson’s correlations were used to generate the 419 × 419 RSFC matrices.

#### 2.1.5 eNKI-RS

The enhanced Nathan Kline Institute-Rockland Sample (eNKI-RS) is a community sample of over 1,000 participants (age 6–85 years), with measures including various physiological and psychological assessments, genetic information, and neuroimaging data (Nooner et al., 2012). We manually selected commonly used cognitive performance measures and behavioral scores with less than 10% of missing value, yielding 61 phenotypes and 896 participants with at least one phenotype.

Details of the fMRI preprocessing can be found in our previous study (Wu et al., 2022), but briefly, eNKI-RS data were pre-processed with fMRIprep (Esteban et al., 2019) with default configuration and additional ICA-AROMA denoising (Pruim, Mennes, Buitelaar, et al., 2015;Pruim, Mennes, van Rooij, et al., 2015). Additional nuisance regression was then performed with regressors corresponding to 24 motion parameters, white matter signal, CSF signal, and their temporal derivatives (Wu et al., 2022). The pre-processed fMRI data in MNI152 space were used to compute 419 × 419 RSFC matrices. Here, the cortical parcels were based on the Schaefer parcellation in MNI152 space, while the subcortical parcels were obtained by FreeSurfer recon-all of the MNI152 template.

#### 2.1.6 HCP-YA

The Human Connectome Project (HCP Young Adult, HCP-YA) contains brain imaging data and phenotypes from healthy young adults (age 22–35 years) (Van Essen et al., 2013). We used 35 phenotypes across cognition, personality, and emotion, consistent with our previous study (He et al., 2022). There are 1,019 participants with all 35 phenotypes in the end.

For the RSFC data, we used ICA-FIX MSMALL time series in the grayordinate (combined surface and subcortical volumetric) fsLR_32k space (Glasser et al., 2013). The time series were averaged within each cortical and subcortical parcel to calculate 419 × 419 RSFC matrices. Here, the cortical parcels were based on the Schaefer parcellation in fsLR space, while the subcortical parcels were defined by the HCP preprocessing pipeline based on FreeSurfer (Glasser et al., 2013).

#### 2.1.7 HCP-Aging

The Human Connectome Project Aging (HCP-Aging) study enrolls 1,500+ healthy adults (age 36–100+ years) (Harms et al., 2018). We manually selected commonly used behavioral measures, resulting in 45 phenotypes and 656 participants with at least one phenotype. The resting-fMRI data after ICA-FIX denoising in MNI152 space were used, following our previous study (Wu et al., 2022). Nuisance regression was then implemented, controlling for 24 motion parameters, white matter signal, CSF signal, and their temporal derivatives (Wu et al., 2022). The time series were averaged within each cortical and subcortical parcel to calculate 419 × 419 RSFC matrices. Here, the cortical parcels were based on the Schaefer parcellation in MNI152 space, while the subcortical parcels were obtained by FreeSurfer recon-all of the MNI152 template.

### 2.2 Data split overview

We split the datasets into a meta-training (source) set and a meta-test (target) set, as shown inFigure 1. For each meta-training dataset, we randomly divided the participants into training and validation sets comprising 80% and 20% of the participants respectively. The training and validation sets are used to train and tune the hyperparameters of one or more “base-learners” to predict corresponding source phenotypes from the meta-training dataset. We note that the splits into training and validation sets were completely random, and no attempt was made to match the demographics (e.g., age and sex) between training and validation sets. Matching demographics between training and validation sets might potentially improve the prediction in the validation sets, but it is unclear whether this would be helpful for the meta-test set, whose demographics might differ from the meta-training sets. In fact, one might even speculate that demographic differences between training and validation sets could help the base-learners to be more robust to demographic differences between meta-training and meta-test datasets.

For each meta-test dataset, there are target phenotypes we want to predict from RSFC. For cross-dataset prediction, we trained a “meta-learner” using K participants in the meta-test dataset (i.e., where K = 10, 20, 50, 100, 200) with observed meta-test phenotypes, which is a setting known as “K-shot learning” (Kadam & Vaidya, 2020). The meta-learner exploits the relationship between source and target phenotypes via the previously trained base-learners from the meta-training datasets, thus transferring knowledge from the meta-training datasets to the meta-test dataset. Finally, we evaluated the prediction performance of meta-test phenotypes on the remaining N – K meta-test participants, using Pearson’s correlation and predictive coefficient of determinant (COD) as metrics.

### 2.3 Prediction approaches

Across all approaches, we vectorized the lower triangular entries of each 419 × 419 RSFC matrix into a feature vector (i.e., 87,571 × 1 vector) to predict phenotypic measures. We note that certain datasets were processed with global signal regression (GSR), while others were processed with ICA-FIX (Table 1). It is well known that GSR centers the distribution of RSFC values at zero (Murphy et al., 2009), which is not the case for ICA-FIX. Therefore, for all cross-dataset algorithms (i.e., all algorithms except kernel ridge regression), we normalized the RSFC vector for each participant independently, by subtracting the mean and then dividing by the L2-norm of the 87,571 × 1 FC vector. Although we did not perform this normalization for classical kernel ridge regression (Section 2.3.1), we note that this normalization has no effect on kernel ridge regression. The reason is that we used the correlation metric to compute the kernel similarity (Section 2.3.1), so Pearson’s correlation between two normalized RSFC matrices will be the same as Pearson’s correlation between two unnormalized RSFC matrices.

Following our previous study (He et al., 2022), statistical difference between algorithms was evaluated using a bootstrapping approach (more details inSupplementary Methods S3). Multiple comparisons were corrected using a false discovery rate (FDR) of q < 0.05. FDR was applied to all K-shots, across all pairs of algorithms and both evaluation metrics (Pearson’s correlation and COD).

#### 2.3.1 Baseline 1: Classical KRR

**Fig. 2.**
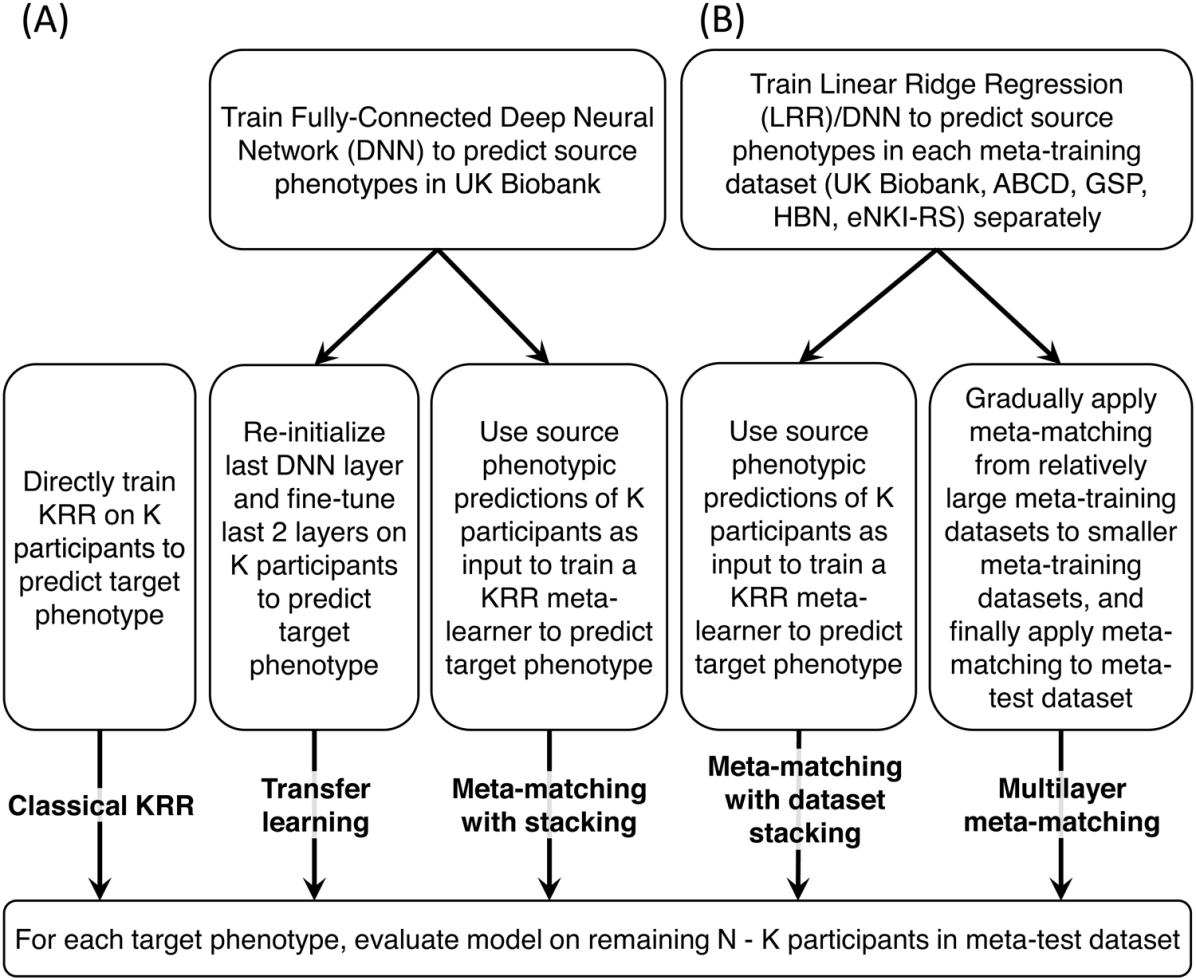
Schematic of different approaches. (A) Schematic of three baselines: classical kernel ridge regression (KRR), transfer learning, and meta-matching with stacking from our previous study (He et al., 2022). (B) Schematic of two proposed approaches: meta-matching with dataset stacking and multilayer meta-matching. Observe the large sample imbalance in the meta-training set with the smallest source dataset comprising 862 participants and the largest source dataset comprising 36,834 participants.

We choose kernel ridge regression (KRR;Fig. 2A) as a baseline algorithm that does not utilize meta-training on the meta-training set. KRR has been shown to be a highly competitive algorithm for MRI prediction of phenotypic measures (He et al., 2020;Kong et al., 2023;Ooi et al., 2022). Consistent with our previous studies, the kernel similarity between participants was defined based on similarity (Pearson’s correlation) between the lower triangular portions of the RSFC matrices. More specifically, the procedure is as follows. Suppose the meta-test dataset has N participants in total. For each target phenotype in the meta-test dataset, we trained a KRR model and tuned the hyper-parameter λ (L2 regularization weight) with 5-fold cross-validation, using K random participants with observed target phenotypes (i.e., K-shot). The optimal λ was then used to train a final KRR model using all K participants. We then evaluated the model performance on the remaining N – K participants using Pearson’s correlation and COD. The procedure was repeated 100 times with a different random set of K participants. The evaluation metrics were averaged across the 100 repetitions to ensure the robustness of the results.

#### 2.3.2 Baseline 2: Transfer learning

As a second baseline, we consider transfer learning (Weiss et al., 2016). As illustrated inFigure 2A, we pre-trained a deep neural network (DNN) in the UK Biobank to simultaneously predict 67 source phenotypes from RSFC (maximum training epochs = 100). The DNN is a simple fully-connected feedforward neural network (also known as a multi-layer perceptron) with 67 output nodes. Rectifying linear units (ReLU) were used as activation functions for all hidden layers. As mentioned inSection 2.2, 80% of the data was used for training and 20% was used for tuning DNN hyper-parameters. The hyper-parameters (e.g., number of layers, number of nodes, learning rate, dropout rate, etc.) were tuned using the Optuna package (Akiba et al., 2019). As a final step, we used 80% of the data for training with the optimal hyperparameters, and the remaining 20% of data for early stopping to reduce the possibility of overfitting. Detailed information about DNN hyper-parameters is found inSupplementary Methods S1.

The pre-trained DNN was then translated using K meta-test participants to predict a target phenotype. Because we are predicting different phenotypes in the meta-test dataset, for a given target phenotype, the last layer of the pre-trained DNN was re-initialized from scratch, and the last two layers of the DNN were then fine-tuned on K random participants with observed target phenotypes (i.e., K-shot). An optimal fixed learning rate was obtained by 5-fold cross-validation and grid search of the K participants. The optimal learning rate was then used to perform fine-tune a final model using all K participants. For both the 5-fold cross-validation and the final round of fine-tuning, the maximum fine-tuning epochs was set to be 10 with 80% of K participants used for training and 20% used to evaluate validation loss for early stopping, to reduce the possibility of overfitting. This final trained model was evaluated in the remaining N – K participants.

#### 2.3.3 Baseline 3: Meta-matching with stacking

The third baseline is the “meta-matching with stacking” algorithm (Fig. 2A) from the original meta-matching study (He et al., 2022). The original study proposed several meta-matching algorithms. Here, we used the stacking approach because it exhibited the best prediction performance in the original study.

Similar to transfer learning, the meta-matching with stacking approach utilized the same pre-trained DNN from the UK Biobank (seeSection 2.3.2). To adapt the DNN to the meta-test dataset, the DNN was applied to the RSFC of the K participants, yielding 67 predictions per participant. The 67 predictions were then used as features to train a KRR model for predicting the target phenotype using the K participants (i.e., stacking;Wolpert, 1992).

The KRR model utilized the correlation kernel, and the KRR hyperparameter λ was tuned using grid search and 5-fold cross-validation on the K participants. The optimal λ was then used to train a final KRR model using all K participants. The prediction performances were evaluated on the remaining N – K participants using Pearson’s correlation and COD as metrics. This procedure was repeated 100 times with a different random sample of K participants.

It is worthwhile highlighting a deviation from the original meta-matching with stacking implementation (He et al., 2022). The original implementation utilized K features for stacking when K < 67. Here, we decided to simply use all 67 features because experimentation after the publication of our previous study (not shown) suggested the constraint was unnecessary.

#### 2.3.4 Meta-matching with dataset stacking

A naive approach to extending meta-matching with stacking to multiple datasets is to train independent prediction model(s) in each meta-training (source) dataset and then “stack” the prediction models based on K participants in the meta-test dataset. We refer to this approach as meta-matching with dataset stacking (Fig. 2B).

For the UK Biobank, we trained a DNN model to predict 67 phenotypes, as well as 67 Linear Ridge Regression (LRR) models to predict 67 phenotypes, to improve prediction performance via ensemble learning (Dietterich, 2000), yielding 67 × 2 = 138 predictions. We note that the original version of our manuscript utilized KRR instead of LRR. However, KRR requires computing the similarity between a test individual’s FC with the training individuals’ FC. The implication is that a researcher applying meta-matching to their own small dataset would require access to the original FC data from the meta-training set, which is undesirable.

We note that the DNN model is identical to the pre-trained DNN from the transfer learning baseline. The remaining four datasets (ABCD, GSP, HBN, eNKI-RS) were a lot smaller than the UK Biobank, so instead of training a DNN, we simply trained an LRR model for each source phenotype and each meta-training dataset. The regularization hyperparameter λ was tuned using grid search and 5-fold cross-validation on the full dataset, and the optimal λ was then used to train a final LRR model using the full dataset. The LRR and DNN models were applied to the RSFC of the K participants (of the meta-test dataset), yielding a total of 67 × 2 + 36 + 23 + 42 + 61 = 296 phenotypic predictions for each participant.

Similar to the meta-matching with stacking approach (Section 2.3.3), the predictions were then used as features to train a KRR model for predicting the target phenotype using the K participants (i.e., stacking). The KRR model utilized the correlation kernel, and the KRR hyperparameter λ was tuned using grid search and 5-fold cross-validation on the K participants. The optimal λ was then used to train a final KRR model using all K participants.

The prediction performances were evaluated on the remaining N – K participants using Pearson’s correlation and COD as metrics. This procedure was repeated 100 times with a different random sample of K participants.

#### 2.3.5 Multilayer meta-matching

As an alternative to “meta-matching with dataset stacking,” we made use of the fact “meta-matching with stacking” can improve the prediction of smaller datasets. Therefore, “multilayer meta-matching” (Fig. 2B) gradually applied meta-matching with stacking from relatively large source datasets (e.g., UK Biobank) to smaller datasets (e.g., GSP, HBN, etc), to generate additional features for a final round of stacking using the K participants from the meta-test dataset.

**Fig. 3.**
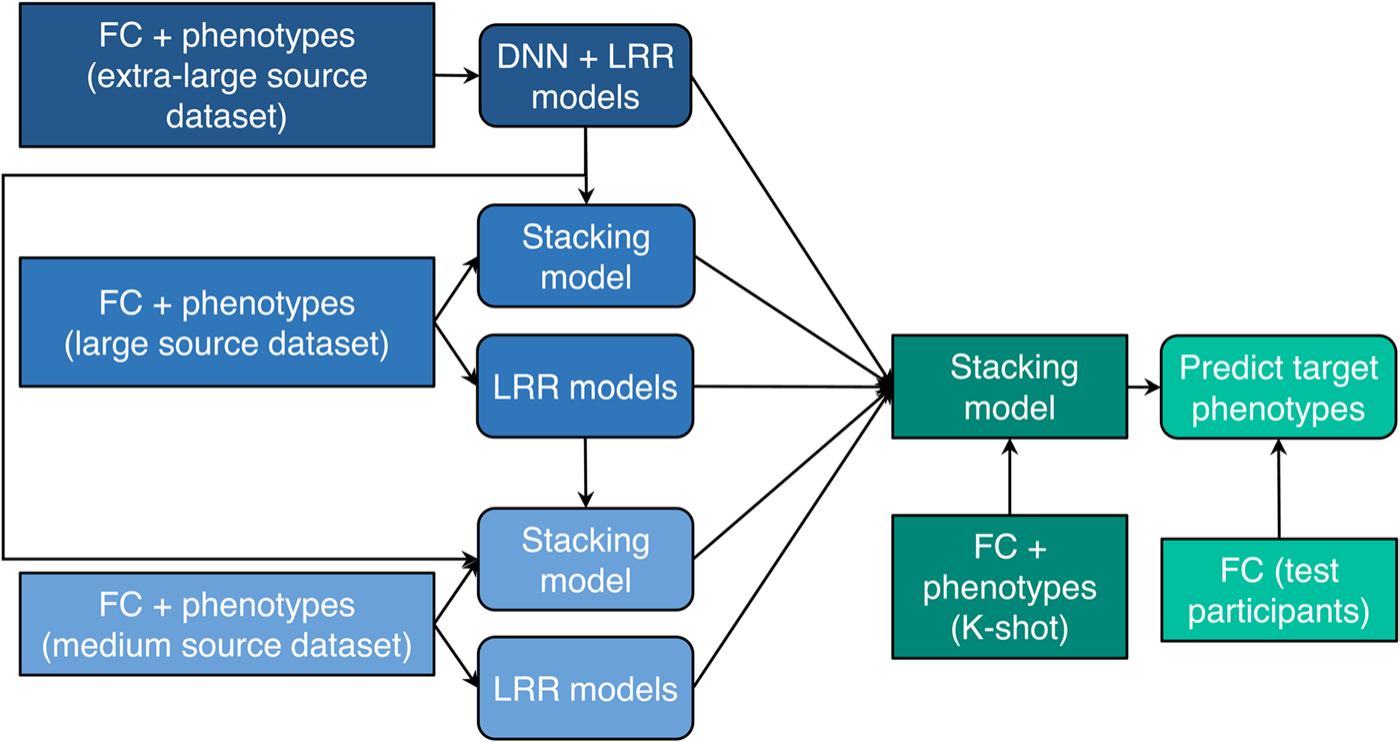
Multilayer meta-matching. We divided source datasets into extra-large (UK Biobank), large (ABCD), and medium (GSP/HBN/eNKI-RS) source datasets. Multi-layer meta-matching gradually applied meta-matching with stacking from relatively large source datasets (e.g., UK Biobank) to smaller datasets (e.g., HCP), to generate additional features for a final round of stacking using the K participants from the meta-test dataset.

In the current study, we instantiated multilayer meta-matching by dividing the meta-training datasets into three groups: extra-large source dataset (comprising only UK Biobank in the current study), large source datasets (comprising only ABCD in the current study), and medium source datasets (comprising GSP, HBN, and eNKI-RS in the current study). Multilayer meta-matching proceeds as follows (Fig. 3).

In the case of the extra-large dataset (UK Biobank), we have previously trained DNN and LRR models to predict 67 phenotypes (Section 2.3.4). The same two models were applied to the K meta-test dataset participants, yielding 67 × 2 = 134 phenotypic predictions, which will be concatenated with the predictions from the other models (below) for stacking.

In the case of the large dataset (ABCD), we have previously trained an LRR model to predict 36 phenotypes in the ABCD dataset (Section 2.3.4). The same model was applied to the K meta-test dataset participants, yielding 36 predictions. Furthermore, the DNN and LRR models from the extra-large dataset (UK Biobank) were also combined to predict the 36 ABCD phenotypes via the meta-matching with stacking procedure (He et al., 2022). The resulting stacking model was applied to the K meta-test dataset participants, yielding 36 predictions. Therefore, models from the ABCD dataset yielded a total of 36 × 2 = 72 phenotypic predictions for each of the K meta-test dataset participants, which will be concatenated with the 134 predictions from the UK Biobank (above) and predictions from the other models (below) for stacking.

Finally, in the case of the medium source dataset (GSP, HBN, or eNKI-RS), let us use the GSP dataset, which had 23 phenotypes, as an example. First, we have previously trained an LRR model to predict 23 phenotypes in the GSP dataset (Section 2.3.4). The same model was applied to the K meta-test dataset participants, yielding 23 predictions. Second, the DNN and LRR models from the extra-large dataset (UK Biobank), as well as the LRR models from the large dataset (ABCD) were also combined to predict the 23 GSP phenotypes via the meta-matching with stacking procedure (He et al., 2022). The resulting stacking model was applied to the K meta-test dataset participants, yielding 23 predictions. Therefore, in total, the GSP dataset contributed 23 × 2 = 46 phenotypic predictions in each of the K meta-test dataset participants. Similarly, the HBN and eNKI-RS datasets contributed 42 × 2 = 84 and 61 × 2 = 122 phenotypic predictions.

Finally, all the phenotypic predictions (134 + 72 + 46 + 84 + 122 = 458) were concatenated and used to train a KRR model on the K meta-test dataset participants (i.e., stacking). Once again, the KRR model utilized the correlation kernel and the KRR hyperparameter λ was tuned using grid search and 5-fold cross-validation on the K participants. The optimal λ was then used to train a final KRR model using all K participants.

The prediction performances were evaluated on the remaining N – K participants using Pearson’s correlation and COD as metrics. This procedure was repeated 100 times with a different random sample of K participants.

It is worth noting that the number of features used by the final stacking procedure was 458 in multilayer meta-matching, compared with 296 features in meta-matching with dataset stacking. More specifically, the number of features directly generated the UK Biobank models is 134 for both approaches. In the case of the large and medium-sized datasets, the number of features are doubled from 36 (ABCD), 23 (GSP), 42 (HBN), and 61 (eNKI-RS) to 72, 46, 84, and 122 respectively. We note that 458 features are still of much lower dimensionality than the raw FC matrices.

### 2.4 Feature importance based on the Haufe transform

Here, we are adapting models pre-trained with different phenotypes to predict new phenotypes in a meta-test dataset with potentially different demographics from the source datasets. A potential concern is that the interpretation of these adapted models (meta-matching or transfer learning models) might be “tainted” by this pre-training. To quantify this bias that might arise from pre-training, we needed to define a ground truth. Here, we assumed that the full HCP-YA and HCP-Aging datasets are sufficiently large, so that a model trained with the full dataset to predict a particular meta-test phenotype will not be biased by the pre-training (since there is no pre-training).

However, what predictive model should be used in this analysis? Since KRR has been shown to be a highly competitive algorithm for MRI prediction of phenotypic measures (He et al., 2020;Kong et al., 2023;Ooi et al., 2022), we decided to train a KRR model on the full HCP-YA (or HCP-Aging) dataset and then applied the Haufe transform to the KRR model to generate pseudo ground truth feature importance weights. The Haufe transform involved computing the covariance between each FC edge and the phenotypic prediction across all participants in the meta-test set (Chen et al., 2022;Haufe et al., 2014). The result is a feature importance value for each RSFC edge. A positive (or negative) feature importance value indicates that higher RSFC for the edge was associated with the prediction model predicting greater (or lower) value for the phenotype.

We chose the Haufe transform because it has been shown to be optimal for linear models (Haufe et al., 2014) and KRR can be reformulated as a linear model of our use of the linear kernel. Furthermore, previous studies have shown that the Haufe transform led to highly-reliable feature importance weights, which are similar across different predictive models (Chen et al., 2023;Tian & Zalesky, 2021), suggesting that our pseudo ground truth will not be sensitive to our choice of KRR as the pseudo ground truth predictive model.

We compared the Haufe transform of the pseudo ground truth with the Haufe transform for each approach (classical KRR, meta-matching, and transfer-learning) for the K = 100 scenario, which involved computing the covariance between each FC edge and the phenotypic prediction across the K participants (Chen et al., 2022;Haufe et al., 2014). We then correlated the resulting feature importance values of each approach with the pseudo ground truth. We repeated this procedure 100 times, and averaged the correlations with the pseudo ground truth across 100 repetitions. Given the relatively small sample (K = 100), we did not expect that meta-matching will yield very similar feature importance values as the pseudo ground truth. However, we hoped that the deviation between our meta-matching models and the pseudo ground truth is not worse than classical KRR (trained on 100 participants).

## 3 Results

### 3.1 Meta-matching with stacking outperformed classical KRR and transfer learning

**Fig. 4.**
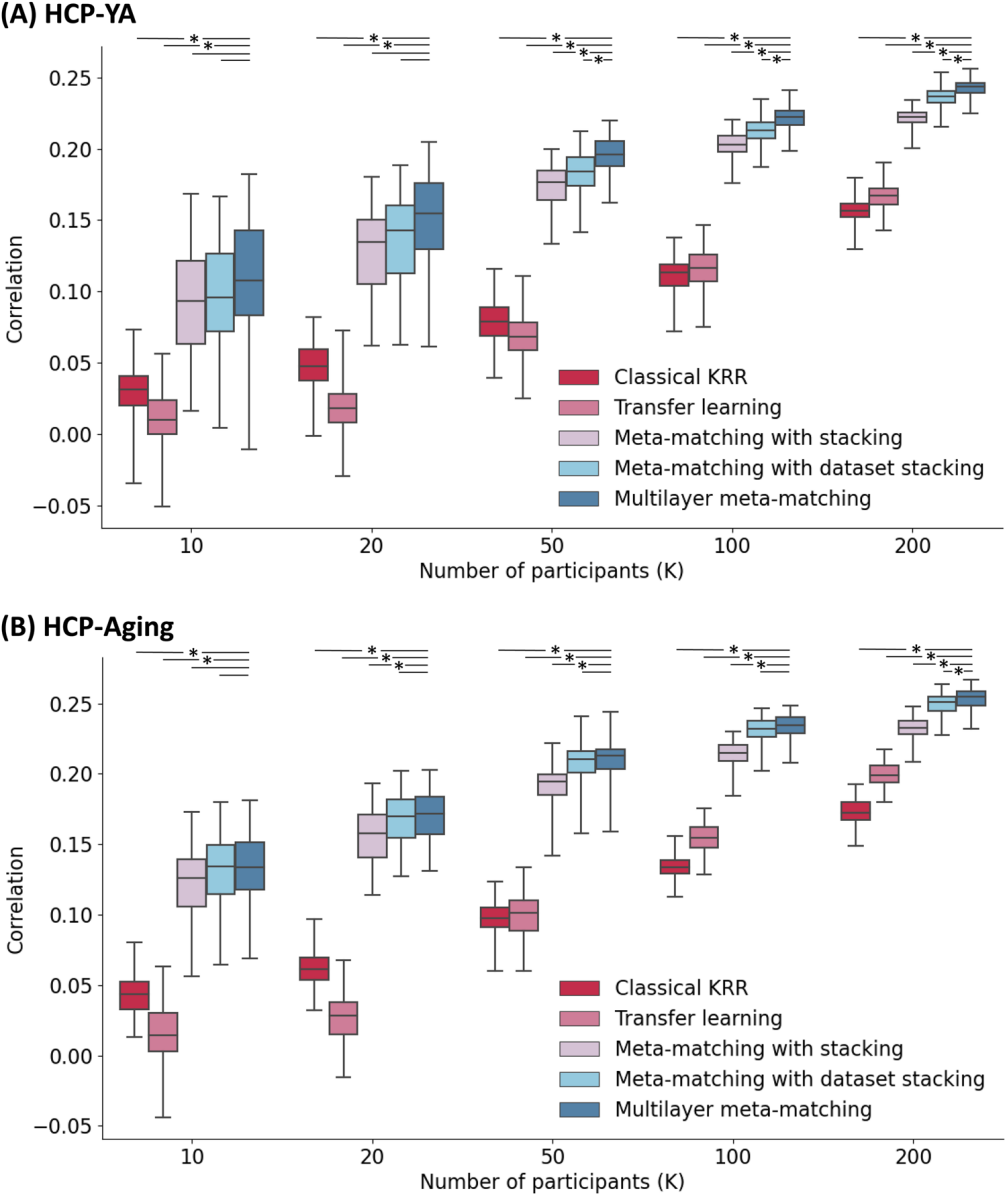
Prediction performance (Pearson’s correlation) in the HCP-YA and HCP-Aging datasets. (A) Phenotypic prediction performance in terms of Pearson’s correlation (averaged across 35 meta-test phenotypes) in the HCP-YA dataset. Horizontal axis is the number of participants in the HCP-YA dataset used to adapt the models trained from the meta-training source datasets. Boxplots represent variability across 100 repetitions of sampling K participants. The bottom and top edges of the box indicate the 25th and 75th percentiles, respectively. Whiskers correspond to 1.5 times the interquartile range. “*” indicates statistical significance between multilayer meta-matching and other approaches (after correction for multiple comparisons with FDR q < 0.05). Dash line without “*” indicates a lack of significance. (B) Same plot as panel A except that the analyses were performed in the HCP-Aging dataset. The full set of p values are reported inTables S9 and S10.

**Fig. 5.**
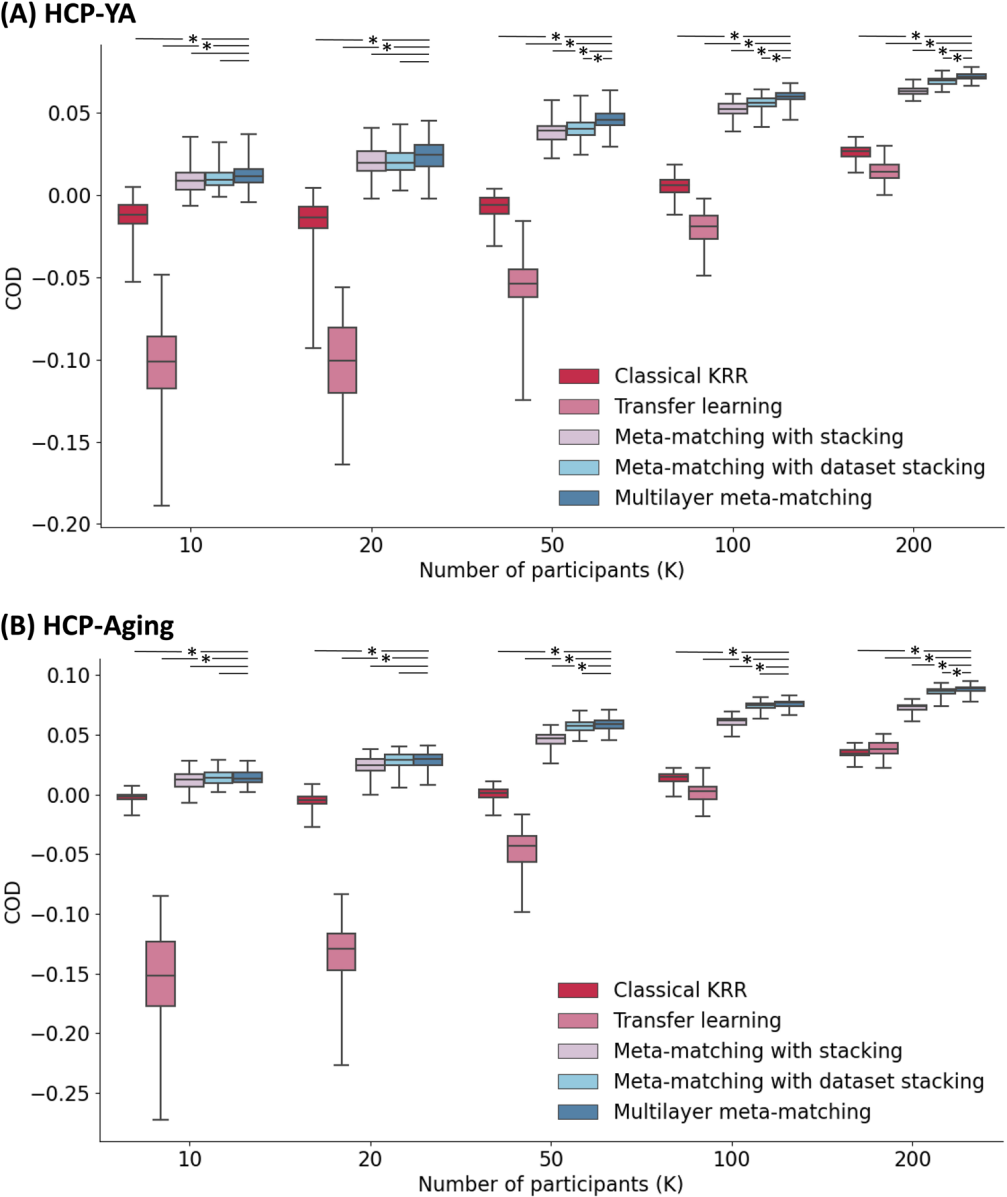
Prediction performance (COD) in the HCP-YA and HCP-Aging datasets. (A) Phenotypic prediction performance in terms of COD (averaged across 35 meta-test phenotypes) in the HCP-YA meta-test set. Horizontal axis is the number of participants in the HCP-YA dataset used to adapt the models trained from the meta-training source datasets. Boxplots represent variability across 100 repetitions of sampling K participants. The bottom and top edges of the box indicate the 25th and 75th percentiles, respectively. Whiskers correspond to 1.5 times the interquartile range. “*” indicates statistical significance between multilayer meta-matching and other approaches (after correction for multiple comparisons with FDR q < 0.05). Dash line without “*” indicates a lack of significance. (B) Same plot as panel A, except that the analyses were performed in the HCP-Aging dataset. The full set of p values are reported inTables S9 and S10.

Figure 4AandBshow the prediction accuracy (Pearson’s correlation coefficient) of various approaches in the HCP-YA and HCP-Aging meta-test datasets respectively. Results were averaged across 35 HCP-YA (or 45 HCP-Aging) phenotypes. The horizontal axis is the number of few-shot participants (K, where K = 10, 20, 50, 100, 200). The vertical axis is Pearson’s correlation of phenotypic prediction. Boxplots represent variability across the 100 repetitions of sampling K participants (i.e., K-shot).Figure 5shows results for COD. Bootstrapping results are shown inFigures S1 and S2, while p values are reported inTables S9 and S10. All bolded p values (Tables S9 and S10) survived an FDR of q < 0.05.

Consistent with our previous study (He et al., 2022), meta-matching with stacking outperformed classical KRR in the HCP-YA dataset (Figs. 4Aand5A;Tables S9). Here, we extended the previous results by showing consistent improvements over KRR in the HCP-Aging dataset. More specifically, in the case of the HCP-YA dataset and K > 10 (Table S9), meta-matching with stacking was statistically better than classical KRR with the largest p < 0.01 across both evaluation metrics (Pearson’s correlation and COD). In the case of HCP-Aging and K > 10 (Table S10), meta-matching with stacking was statistically better than classical KRR with the largest p < 0.002 across both evaluation metrics.

Furthermore, meta-matching with stacking also outperformed transfer learning across both datasets (Figs. 4Aand5A). In the case of the HCP-YA dataset and K ≥ 10 (Table S9), meta-matching with stacking was statistically better than transfer learning with p values <0.02 across both evaluation metrics (Pearson’s correlation and COD). In the case of HCP-Aging and K ≥ 10 (Table S10), meta-matching with stacking was statistically better than transfer learning with the largest p < 0.001 across both evaluation metrics.

Interestingly, transfer learning performed consistently worse than classical KRR for K < 50, especially for the COD metric (Figs. 4Aand5A).

### 3.2 Improvement from additional meta-training source datasets

By including additional meta-training datasets, meta-matching with dataset stacking and multilayer meta-matching were numerically better than meta-matching with stacking (which only utilized the UK Biobank) for almost all values of K (Figs. 4and5).

In the case of the HCP-YA dataset and K > 100 (Table S9), meta-matching with dataset stacking was statistically better than meta-matching with stacking with the largest p < 0.001 across both evaluation metrics (Pearson’s correlation and COD). In the case of the HCP-Aging and K > 20 (Table S10), meta-matching with dataset stacking was statistically better than meta-matching with stacking with the largest p < 0.001 across both evaluation metrics.

On the other hand, in the case of the HCP-YA dataset and K > 20 (Table S9), multilayer meta-matching was statistically better than meta-matching with stacking with the largest p < 0.03 across both evaluation metrics. In the case of the HCP-Aging and K > 20 (Table S10), multilayer meta-matching was statistically better than meta-matching with stacking with the largest p < 0.001 across both evaluation metrics.

We observe that the p values for multilayer meta-matching were generally stronger (i.e., smaller) than meta-matching with dataset stacking and will directly compare the two meta-matching variants in the next section.

### 3.3 Multilayer meta-matching modestly outperformed meta-matching with dataset stacking

Multi-layer meta-matching was numerically better than meta-matching with dataset stacking for almost all values of K. This improvement was significant for larger values of K. In the case of the HCP-YA dataset and K > 20 (Table S9), multi-layer meta-matching was statistically better than meta-matching with dataset stacking with the largest p < 0.01 for both evaluation metrics (correlation and COD). For HCP-Aging, multilayer meta-matching was statistically better than meta-matching with dataset stacking for K = 200 for both evaluation metrics (p < 0.03;Table S10). Overall, the results suggest that multilayer meta-matching was modestly more effective than meta-matching with dataset stacking.

In the introduction, we suggested that since meta-matching with stacking (He et al., 2022) improved prediction significantly in small datasets, by applying the original meta-matching with stacking to the smaller datasets, the resulting features might be more helpful for the final stacking procedure, compared with just training KRR models in the smaller datasets directly. To test this hypothesis, we performed 5-fold cross-validation on three medium datasets (i.e., GSP, HBN, and eNKI-RS), to predict phenotypes using classical KRR. We note that the KRR models are used by the meta-matching with dataset stacking approach in the meta-test set. We also performed 5-fold cross-validation on the medium datasets using meta-matching with stacking based on the DNN and LRR models from the UK Biobank and the LRR models from ABCD. We find that meta-matching with stacking yielded better prediction performance than the KRR models in two of the three datasets (Table 2), thus providing some support for our hypothesis.

**Table 2.** Prediction using classical KRR versus meta-matching with stacking on medium source datasets.

Datasets	Prediction performance (Pearson’s correlation) of classical KRR	Prediction performance of (Pearson’s correlation) of meta-matching w/ stacking (from UKBB + ABCD)	Correlation between phenotypic prediction by above two methods
GSP	0.0953	0.106	0.400
HBN	0.167	0.144	0.433
eNKI-RS	0.154	0.196	0.600

In the same analysis, we found that phenotypic predictions from classical KRR and meta-matching with stacking are not strongly correlated (r = 0.4 to 0.6;Table 2). We remind the reader that the predictions of these models are used as features for stacking in the meta-test set. Since ensembles of diverse machine-learning models lead to better prediction performance (Kuncheva & Whitaker, 2003), we speculate that the more diverse predictions utilized by multilayer meta-matching might lead to better prediction performance than meta-matching with dataset stacking.

### 3.4 Different improvements on different phenotypes by multilayer meta-matching

**Fig. 6.**
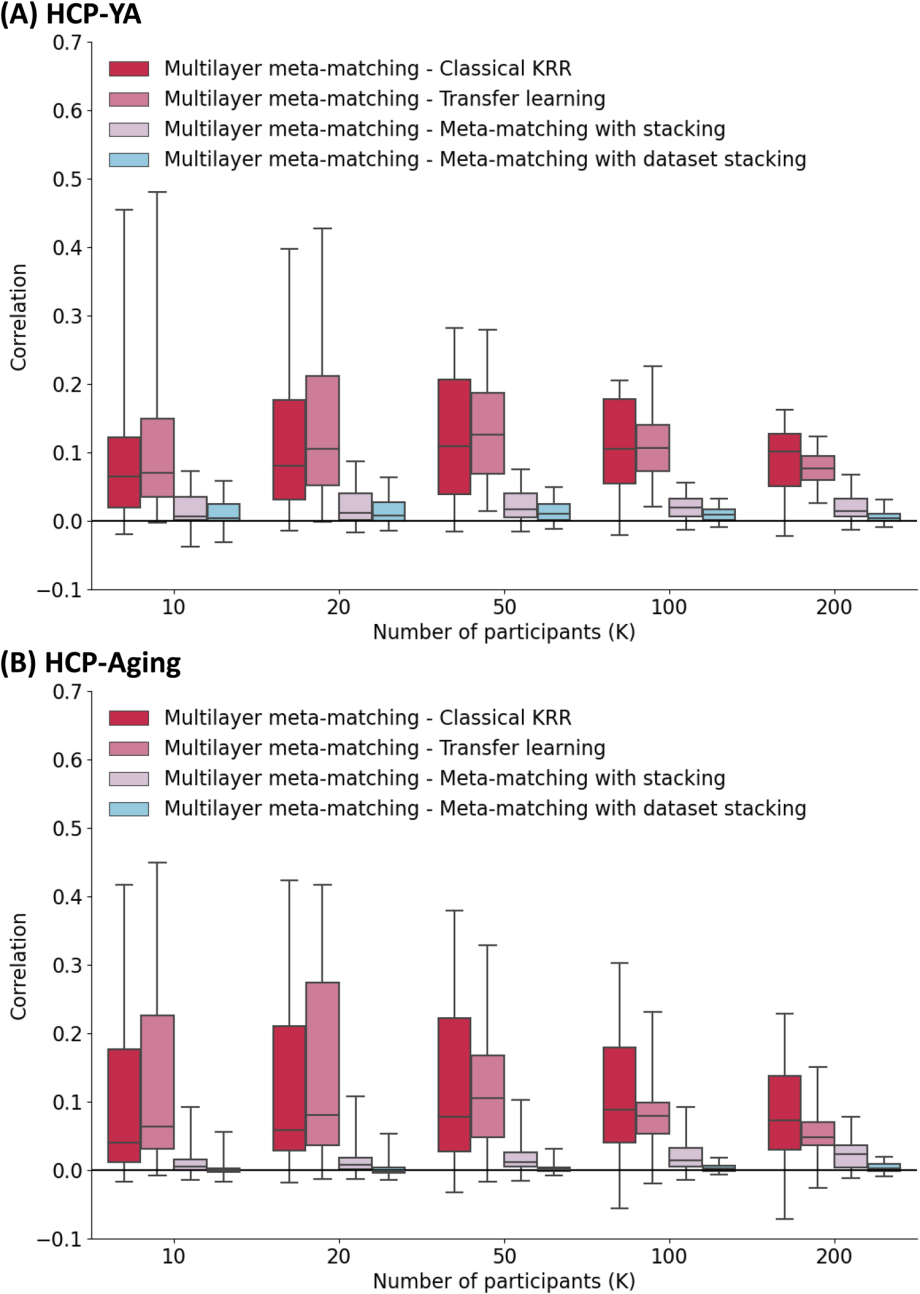
Numerical improvement in prediction performance (Pearson’s correlation) across different phenotypes in the HCP-YA and HCP-Aging datasets. (A) Phenotypic prediction performance (averaged across 100 repetitions of sampling K participants) in the HCP-YA dataset. Horizontal axis is the number of participants in the HCP-YA dataset used to adapt the models trained from the meta-training datasets. Boxplots represent variability across the 35 HCP-YA phenotypes. The bottom and top edges of the box indicate the 25th and 75th percentiles, respectively. Whiskers correspond to 1.5 times the interquartile range. (B) Same plot as panel A except that the analyses were performed in the HCP-Aging dataset with 45 phenotypes.

Figure 6shows the numerical improvement in prediction performance (Pearson’s correlation) of multilayer meta-matching over the other approaches across different phenotypes. The corresponding plot for COD is shown inFigure S3.Table 3shows the percentage of phenotypes in which multilayer meta-matching exhibits numerical improvement in prediction performance (Pearson’s correlation) over other approaches. COD results are shown inTable S11. Compared with classical KRR, transfer learning, and meta-matching with stacking, we found that multilayer meta-matching exhibited numerical improvement for a vast majority of the phenotypes (Table 3;Table S11).

**Table 3.** Percentages of phenotypes with numerical improvement in prediction performance (Pearson’s correlation).

Datasets	K	Multilayer MM vs. classical KRR	Multilayer MM vs. transfer learning	Multilayer MM vs. MM w/ stacking	Multilayer MM vs. MM w/ dataset stacking
HCP-YA	10	85.7%	94.3%	80.0%	74.3%
	20	85.7%	100%	77.1%	74.3%
	50	88.6%	100%	85.7%	80.0%
	100	91.4%	100%	85.7%	77.1%
	200	97.1%	100%	85.7%	74.3%
HCP-Aging	10	86.7%	95.6%	75.6%	44.4%
	20	86.7%	95.6%	77.8%	42.2%
	50	88.9%	93.3%	82.2%	57.7%
	100	88.9%	95.6%	77.8%	57.7%
	200	88.9%	95.6%	82.2%	64.4%

**Fig. 7.**
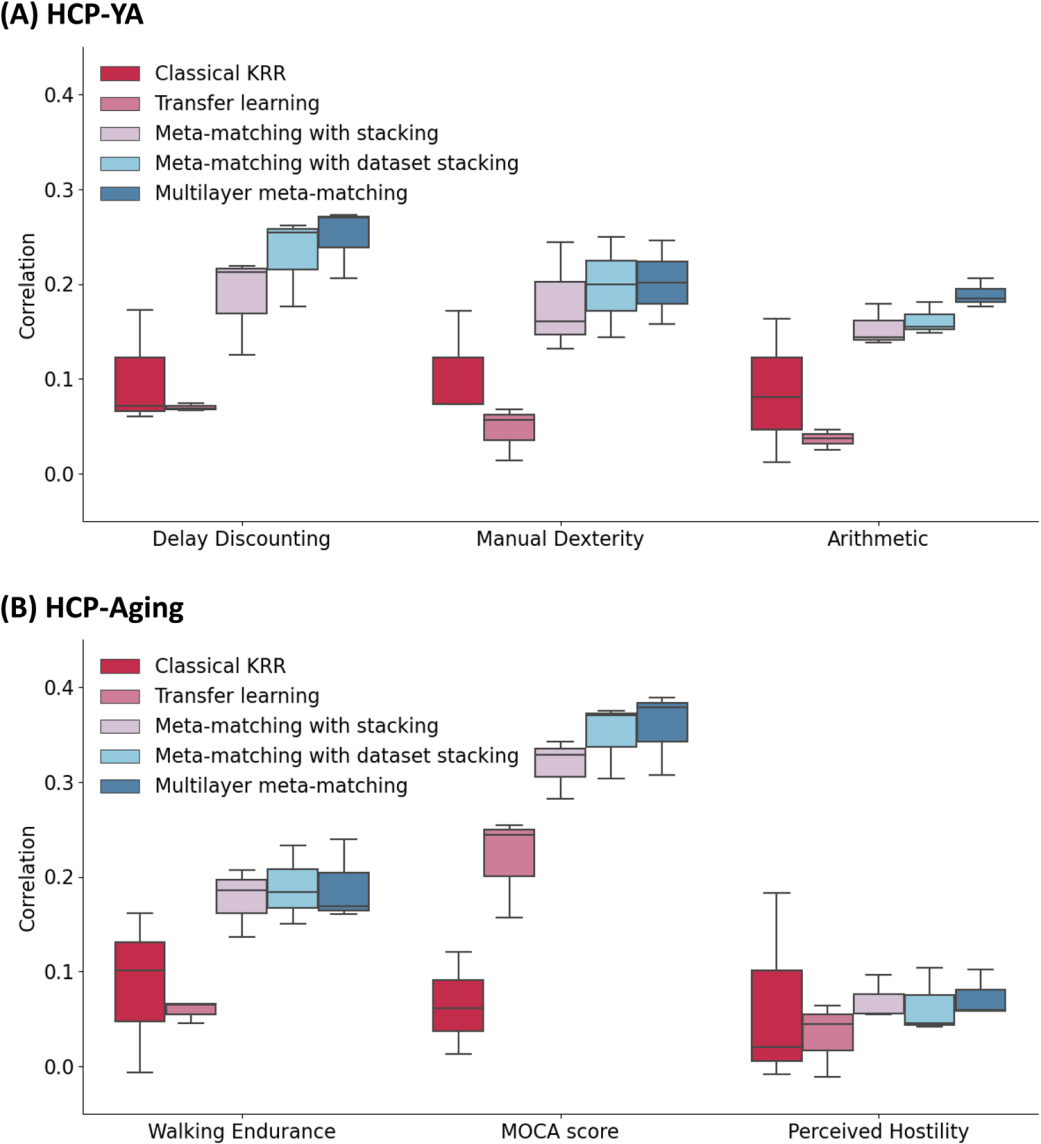
Examples of phenotypic prediction performance in the (A) HCP-YA and (B) HCP-Aging datasets in the case of 100-shot learning (K = 100). Here, prediction performance was measured using Pearson’s correlation. For each box plot, the horizontal line indicates the median. The bottom and top edges of the box indicate the 25th and 75th percentiles, respectively. Whiskers correspond to 1.5 times the interquartile range.

Figure 7illustrates the 100-shot prediction performance (Pearson’s correlation coefficient) of three example meta-test phenotypes across all approaches in the HCP-YA (Fig. 7A) and HCP-Aging (Fig. 7B) datasets. For three illustrated HCP-YA phenotypes (“Delay Discounting,” “Manual Dexterity,” “Arithmetic”), multilayer meta-matching exhibited numerically the best results. On the other hand, among the three illustrated HCP-Aging phenotypes, multilayer meta-matching was numerically worse than meta-matching with stacking and meta-matching with dataset stacking in the case of “Walking Endurance,” but was numerically the best for “MOCA score” and “Perceived Hostility.”

Tables S12 to S15report the numerical improvement of multilayer meta-matching over other baselines for all HCP-YA and HCP-Aging phenotypes (in the 100-shot scenario). In the HCP-YA dataset, multilayer meta-matching was numerically better than classical KRR for many cognitive measures, but also some non-cognitive measures, such as strength and endurance (Tables S12 and S13). This was also the case for the HCP-Aging dataset, and interestingly the phenotype enjoying the greatest improvement was strength (Tables S14 and S15).

### 3.5 Feature importance using the Haufe transform

As shown inFigure 8, across both HCP-YA and HCP-Aging datasets, feature importance values of multilayer meta-matching and classical KRR were equally similar to the pseudo ground truth feature importance values. On the other hand, feature importance values from transfer learning were the most different from the pseudo ground truth. If we only focused on the transfer learning and meta-matching models, we observed a trend in increasing agreement with pseudo ground truth, which parallels the prediction accuracy increase from transfer learning to meta-matching with stacking to meta-matching with dataset stacking and then to multilayer meta-matching.

**Fig. 8.**
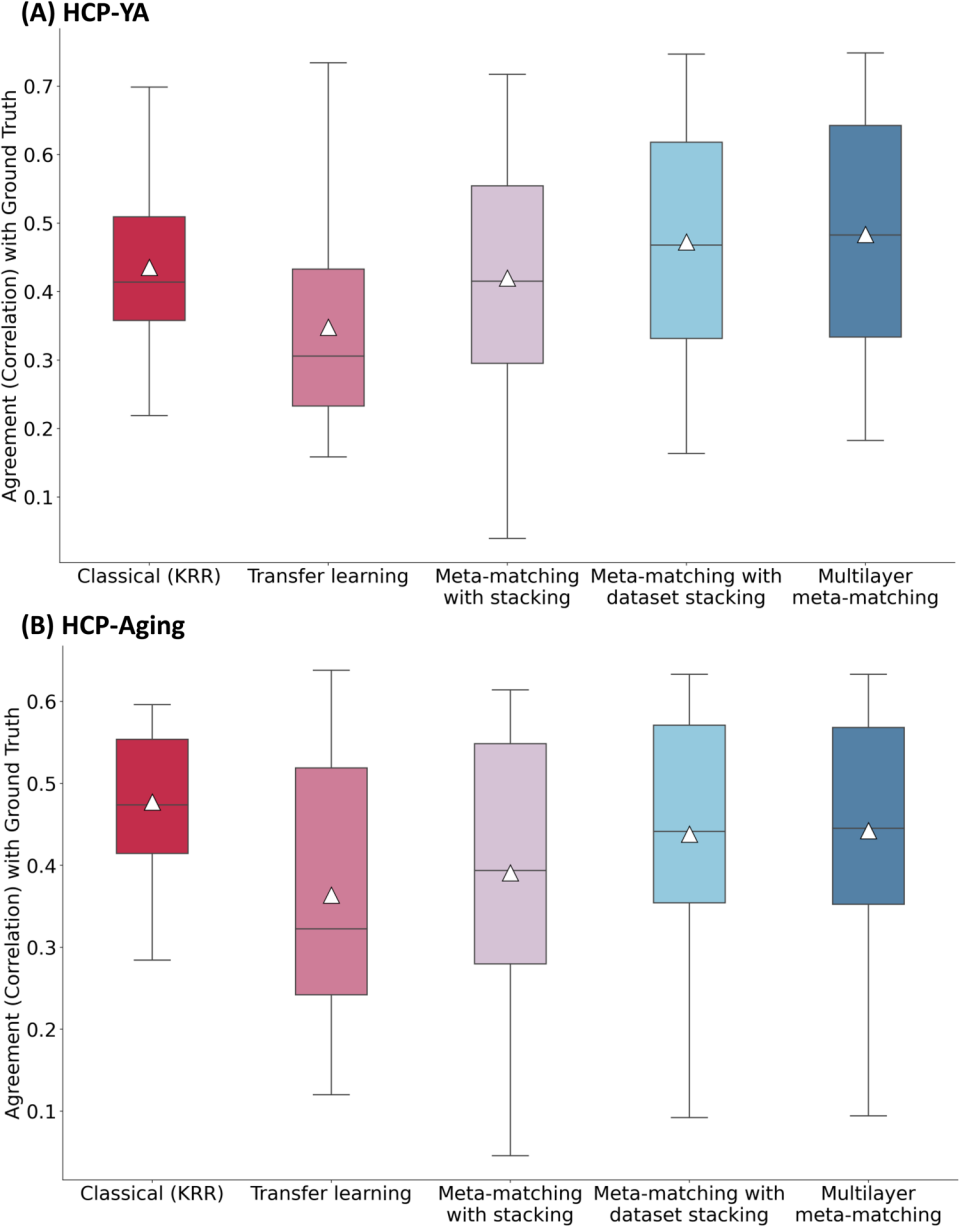
Agreement (correlation) of feature importance values with pseudo ground truth in the (A) HCP-YA and (B) HCP-Aging datasets. For each approach, the Haufe transform was used to estimate feature importance in the 100-shot scenario (K = 100), which was then compared with the pseudo ground truth. Pseudo ground truth feature importance was generated by applying the Haufe transform to a KRR model trained from the full target dataset. For each box plot, the horizontal line indicates the median, and the triangle indicates the mean. The bottom and top edges of the box indicate the 25th and 75th percentiles, respectively. Whiskers correspond to 1.5 times the interquartile range.

## 4 Discussion

In this study, we proposed two meta-matching algorithms to translate phenotypic prediction models from source datasets with disparate sizes to predict new phenotypes in small datasets. Both approaches outperformed meta-matching using a single source dataset (UK Biobank). Both approaches also outperformed classical KRR and classical transfer learning by a big margin. Furthermore, multilayer meta-matching compared favorably with meta-matching with dataset stacking across both HCP-YA and HCP-Aging datasets. In terms of feature importance based on the Haufe transform, we found that feature importance values of multilayer meta-matching and classical KRR seemed to be equally similar to the pseudo ground truth, while feature importance values of transfer learning were the furthest away from the pseudo ground truth. Therefore, there was no trade-off between prediction accuracy and feature importance accuracy (with respect to the pseudo ground truth), which is consistent with our previous study (Chen et al., 2023).

The relatively poor performance of classical transfer learning was somewhat surprising but probably indicated the difficulty of finetuning so many parameters in the very small sample regime. We note that the transfer learning baseline is similar to a meta-matching variant “meta-matching finetune” from our previous study (He et al., 2022), except for one key difference. Both meta-matching finetune and classical transfer learning finetuned the last two layers of the DNN. However, transfer learning initialized the last layer of the DNN from scratch (Section 2.3.2), and then finetuned the last two layers. On the other hand, meta-matching finetune first selected the output node that predicted the K meta-test participants the best (for a particular meta-test phenotype), and retained the weights leading to the output node. The last two layers of the DNN were then finetuned, given that meta-matching finetune was much better than classical KRR (He et al., 2022), but classical transfer learning was worse than KRR in the current study. This further supported the importance of the meta-matching idea.

### 4.1 Meta-learning, transfer learning, and related problems

We mentioned in the introduction that the name “meta-matching” was motivated by the “matching” of meta-training and meta-test phenotypes. The name “meta-matching” was also motivated by its close links with meta-learning (Andrychowicz et al., 2016;Fei-Fei et al., 2006;Finn et al., 2017;Ravi & Larochelle, 2016;Vanschoren, 2019). Meta-learning is often referred to as “learning to learn” and is closely related to “transfer learning” (Hospedales et al., 2021). Both meta-learning and transfer learning seek to improve prediction in a new domain with limited training data using knowledge gained from previous domains (Pan & Yang, 2009). The goal of learning from limited training data (e.g., K training examples) is often referred to as few-shot (or K-shot) learning (Hospedales et al., 2021).

Meta-learning typically involves two learning levels (Huisman et al., 2021). At one level, the algorithm seeks to rapidly learn a new task with limited quantity of data. This rapid learning of a new task is made possible by knowledge learned from earlier tasks at another level. Therefore, meta-matching is similar in spirit to meta-learning. At one level, meta-matching involves training models to predict meta-training phenotypes. These trained models are then rapidly adapted to predict a new meta-test set at another level. However, our meta-matching approach differs from modern meta-learning algorithms that typically involve a meta-objective that is used to optimize an inner-loop learner (Hospedales et al., 2021).

Transfer learning can be broadly defined as using past experience from one or more source tasks to improve learning on a target task (Hospedales et al., 2021). Therefore, meta-learning is one approach that can be used to improve transfer learning (Hospedales et al., 2021). Consequently, we can also consider meta-matching as a type of transfer learning algorithm. One distinction between meta-learning and transfer learning is that meta-learning always involves training a machine-learning model from a wide range of meta-training tasks and then adapting to perform a new prediction problem in the target dataset. On the other hand, in transfer learning, the prediction problem in the target dataset can be the same (Chen et al., 2020;Vakli et al., 2018;Zhang & Bellec, 2020) or different (Hon & Khan, 2017;Lu et al., 2021;Schirmer et al., 2021) in the source dataset. While the prediction problem is the same in the target and source domains, the input feature distribution might be different between the two domains, which is a problem known as domain shift (Hospedales et al., 2021). Domain adaptation is, therefore, a type of transfer learning which seeks to address the problem of domain shift.

Finally, we note that the stacking procedure employed by multilayer meta-matching (and other meta-matching variants) utilized the predictions of meta-training phenotypes as input features to predict new meta-test phenotypes. This contrasts with many RSFC-based prediction approaches that utilized the RSFC data directly (Finn et al., 2015;He et al., 2020), and is reminiscent of studies predicting a phenotype from previously predicted measures (Gal, Tik, et al., 2022;Yoo et al., 2022).

### 4.2 Limitations and future work

One important limitation of meta-matching is that the magnitude of prediction improvement heavily depends on the correlations between meta-training and meta-test phenotypes (He et al., 2022). Consequently, we do not expect all meta-test phenotypes to benefit from meta-matching (Fig. 6). However, it is important to note that this limitation exists for all meta-learning and transfer learning algorithms (Jose & Simeone, 2021;Zhang et al., 2017). Model transfer is easier if the source and target domains are more similar. Performance will degrade if the source and target domains are very different. This observation motivates the addition of more source datasets.

Based on the current trends (Figs. 4and5), we might expect multilayer meta-matching to remain better than classical KRR beyond 200 participants. However, we would expect classical KRR to catch up for larger K, and might ultimately be better than multilayer meta-matching for relatively large K. A hint of this crossover can be found inTable 2, where KRR was numerically better than meta-matching with stacking for 5-fold cross-validation of HBN (N = 930), but numerically worse than meta-matching with stacking for 5-fold cross-validation of GSP (N = 862) and eNKI-RS (N = 896).

Finally, we note that there are multiple possible extensions to the current work. Within the context of resting-state functional connectivity, we could explore the use of individual-specific parcellations, which have been shown to improve phenotypic prediction performance compared with group-level parcellations (Kong et al., 2021;M. Li et al., 2019). Furthermore, previous studies have suggested that other FC measures (e.g., partial correlations) can lead to better prediction performance than Pearson’s correlation (Dadi et al., 2019;Farahibozorg et al., 2021;Pervaiz et al., 2020). Some studies have suggested that fine-grained FC might capture additional behavioral information (Feilong et al., 2021). Therefore, meta-matching models based on other FC measures (e.g., fine-grained FC and partial correlations) might also be explored.

Beyond resting-state functional connectivity, meta-matching can be applied to other imaging modalities, such as task-FC (Chen et al., 2022;Greene et al., 2018) and fMRI during naturalistic stimulus (Finn, 2021;Finn & Bandettini, 2021;Gal, Coldham, et al., 2022), which have shown improvements over RSFC for phenotypic prediction. However, developing meta-matching models for task-fMRI and naturalistic-FC is more challenging because large datasets with consistent task or movie paradigm are not common. Other alternative modalities include anatomical T1 images and diffusion MRI. In the case of anatomical T1 images, we could simply replace the fully connected feedforward DNN used in the current study with 3D convolutional neural networks (Wulan et al., 2024). Finally, the datasets in the current study comprised relatively healthy participants. Meta-matching might be potentially useful for psychiatric populations (Chopra et al., 2022). Including psychiatric datasets to the base model training might further improve generalization to new datasets by increasing the diversity of the source datasets.

## Supplementary Material

Supplementary Material

## Data Availability

This study utilized publicly available data from the UK Biobank (https://www.ukbiobank.ac.uk/), ABCD (https://abcdstudy.org), GSP (http://neuroinformatics.harvard.edu/gsp/), HBN (https://fcon_1000.projects.nitrc.org/indi/cmi_healthy_brain_network), eNKI-RS (http://fcon_1000.projects.nitrc.org/indi/enhanced/), and HCP (https://www.humanconnectome.org/). Data can be accessed via data use agreements. Code for the classical KRR baseline and meta-matching algorithms can be found here (https://github.com/ThomasYeoLab/CBIG/tree/master/stable_projects/predict_phenotypes/Chen2024_MMM). The trained models for multilayer meta-matching are also publicly available (https://github.com/ThomasYeoLab/Meta_matching_models/tree/main/rs-fMRI/v2.0). The code was reviewed by two co-authors (LA and CZ) before merging into the GitHub repository to reduce the chance of coding errors.
