# Supplementary material for: Multilayer meta-matching: Translating phenotypic prediction models from multiple datasets to small data
Source: Imaging Neurosci (Camb). 2024 Jul 17;2:imag-2-00233. doi: 10.1162/imag_a_00233 (PMC12272238; doi:10.1162/imag_a_00233)
Supplement: Supplementary Material [file imag_a_00233-supp.pdf]

Supplementary Materials

This supplementary material is divided into Supplementary Methods and Supplementary Results to complement the Methods and Results sections in the main text, respectively.

Supplementary Methods

Section S1 provides details about DNN used for transfer learning and meta-matching methods. Section S2 provides information about phenotypes used in each dataset.

S1. Details of DNN trained on UK Biobank

In this section, we provide further details about the DNN trained on the UK Biobank, which was used in transfer learning, meta-matching with stacking, meta-matching with dataset stacking and multilayer meta-matching. We used mean square error (MSE) as the loss function. The output layer of DNN has 67 nodes (which is the number of UK Biobank phenotypes). The maximum training epoch was set to 100. The learning rate decreases by half if validation loss has not decreased for 25 epochs. The epoch with the best COD on the validation set was chosen as the optimal epoch. Table S1 shows the final hyperparameters values of the DNN trained on UK Biobank datasets, which were automatically tuned using Optuna (Akiba et al., 2019) with 200 trials.

**Hyper-parameters**	**Values**
Number of layers	4
Number of nodes in hidden layers	512 – 256 – 128
Dropout rate	0.4
Starting learning rate	0.036612177992895435
Weight decay rate	7.321529434575473e-06
Batch size	128

Table S1. Final DNN hyper-parameters estimated by the Optuna package.

S2. Details of phenotypes selected from each dataset

The phenotypes selected for use in UK Biobank, ABCD, GSP, HBN, eNKI-RS, HCP-YA, and HCP-Aging datasets are shown in Table S2 – Table S8 respectively.

Label	Description
Alcohol 1	average monthly spirits intake
Alcohol 2	average weekly champagne plus white wine intake
Alcohol 3	average weekly beer plus cider intake
Time walk	number of days walked 10+ minutes per week
Time drive	time spent driving per day
Time TV	time spent watching television (tv) per day
Sleep	sleep duration per day
Age edu	age completed full time education
Work	weekly length of working hour for main job
Travel	frequency of travelling from home to job workplace per week
#household	number of people in household
Neuro	neuroticism score
Hearing	hearing signal-to-noise-ratio (snr) of triplet (left)
Fluid Int.	fluid intelligence
Match	pairs matching
Sex	sex
Match-o	pairs matching online
Age	age
Trail-o C1	trail making online principal component 1
Trail-o C4	trail making online principal component 4
Digit-o C1	symbol digit substitution online principal component 1
Digit-o C6	symbol digit substitution online principal component 6
Sex G C1	genotype sex inference principal component 1
Sex G C2	genotype sex inference principal component 2
Genetic C1	genetic principal components and heterozygosity principal component 1
Cancer C1	cancer principal component 1
Urine C1	urine assays principal component 1
Blood C2	blood assays principal component 2
Blood C3	blood assays principal component 3
Blood C4	blood assays principal component 4
Blood C5	blood assays principal component 5
Deprive C1	multiple deprivation principal component 1
Dur C1	process durations principal component 1
Dur C2	process durations principal component 2
Dur C4	process durations principal component 4
Trail C1	trail making principal component 1

Tower C1	tower rearranging principal component 1
Digit 1	symbol digit substitution principal component 1
RT C1	reaction time principal component 1
ProMem C1	prospective memory principal component 1
#Mem C1	numeric memory principal component 1
Matrix C1	matrix pattern completion principal component 1
Matrix C2	matrix pattern completion principal component 2
Matrix C3	matrix pattern completion principal component 3
Illness C1	non-cancer illness principal component 1
Illness C4	non-cancer illness principal component 4
Loc C1	location principal component 1
Breath C1	spirometry principal component 1
Grip C1	hand grip strength principal component 1
ECG C1	ECG measures principal component 1
ECG C2	ECG measures principal component 2
ECG C3	ECG measures principal component 3
ECG C6	ECG measures principal component 6
Carotid C1	carotid ultrasound principal component 1
Carotid C5	carotid ultrasound principal component 5
Bone C1	bone-densitometry of heel principal component 1
Bone C3	bone-densitometry of heel principal component 3
Body C1	anthropometry principal component 1
Body C2	anthropometry principal component 2
Body C3	anthropometry principal component 3
BP eye C2	blood pressure & eye measures component 2
BP eye C3	blood pressure & eye measures principal component 3
BP eye C4	blood pressure & eye measures principal component 4
BP eye C5	blood pressure & eye measures principal component 5
BP eye C6	blood pressure & eye measures principal component 6
Family C1	family history (parent's age) principal component 1
Smoke C1	smoke principal component 1

Table S2. Dictionary of 67 phenotypes in the UK Biobank.

ABCD field	Description
cbcl_scr_syn_anxdep_r	AnxDep CBCL Syndrome Scale (raw score)
cbcl_scr_syn_withdep_r	WithDep CBCL Syndrome Scale (raw score)
cbcl_scr_syn_somatic_r	Somatic CBCL Syndrome Scale (raw score)
cbcl_scr_syn_social_r	Social CBCL Syndrome Scale (raw score)
cbcl_scr_syn_thought_r	Thought CBCL Syndrome Scale (raw score)
cbcl_scr_syn_attention_r	Attention CBCL Syndrome Scale (raw score)
cbcl_scr_syn_rulebreak_r	RuleBreak CBCL Syndrome Scale (raw score)
cbcl_scr_syn_aggressive_r	Aggressive CBCL Syndrome Scale (raw score)
nihtbx_picvocab_uncorrected	NIH Toolbox Picture Vocabulary Test Age 3+ v2.0 Uncorrected Standard Score
nihtbx_flanker_uncorrected	NIH Toolbox Flanker Inhibitory Control and Attention Test Ages 8-11 v2.0 Uncorrected Standard Score
nihtbx_list_uncorrected	NIH Toolbox List Sorting Working Memory Test Age 7+ v2.0 Uncorrected Standard Score
nihtbx_cardsort_uncorrected	NIH Toolbox Dimensional Change Card Sort Test Ages 8-11 v2.0 Uncorrected Standard Score
nihtbx_pattern_uncorrected	NIH Toolbox Pattern Comparison Processing Speed Test Age 7+ v2.0 Uncorrected Standard Score
nihtbx_picture_uncorrected	NIH Toolbox Picture Sequence Memory Test Age 8+ Form A v2.0 Uncorrected Standard Score
nihtbx_reading_uncorrected	NIH Toolbox Oral Reading Recognition Test Age 3+ v2.0 Uncorrected Standard Score
nihtbx_fluidcomp_uncorrected	Cognition Fluid Composite Uncorrected Standard Score
nihtbx_cryst_uncorrected	Crystallized Composite Uncorrected Standard Score
nihtbx_totalcomp_uncorrected	Cognition Total Composite Score Uncorrected Standard Score
upps_y_ss_negative_urgency	UPPS-P for Children Short Form, Negative Urgency
upps_y_ss_lack_of_planning	UPPS-P for Children Short Form, Lack of Planning
upps_y_ss_sensation_seeking	UPPS-P for Children Short Form, Sensation Seeking
upps_y_ss_positive_urgency	UPPS-P for Children Short Form, Positive Urgency
upps_y_ss_lack_of_perseverance	UPPS: Lack of Perseverance (GSSF)
bis_y_ss_bis_sum	BIS/BAS: BIS Sum
bis_y_ss_bas_rr	BIS/BAS: BAS Reward Responsiveness
bis_y_ss_bas_drive	BIS/BAS: BAS drive
bis_y_ss_bas_fs	BIS/BAS: BAS Fun Seeking
pps_y_ss_number	Prodromal Psychosis Scale: Number of Yes Responses Sum
pps_y_ss_severity_score	Prodromal Psychosis: Severity Score Sum
pgbi_p_ss_score	Parent General Behavior Inventory SUM
pea_ravlt_sd_trial_vi_tc	RAVLT Short Delay Trial VI Total Correct

pea_ravlt_ld_trial_vii_tc	RAVLT Long Delay Trial VII Total Correct
pea_wiscv_trs	WISC-V Matrix Reasoning Total Raw Score
lmt_scr_perc_correct	Percentage correct of all 32 presented trials
lmt_scr_rt_correct	Average reaction for correct trials
lmt_scr_efficiency	LMT Efficiency

Table S3. Dictionary of 36 phenotypes in the ABCD study.

GSP field	Description
Flanker_S_CORRpc	Flanker Accuracy
Average of MenRot_80_CORRpc, MenRot_120_CORRpc, and MentRot_160_CORRpc	Mental Rotation Accuracy
Shipley_Vocab_Raw	Shipley Vocabulary
Matrix_WAIS	WAIS – Matrix Reasoning
STAI_tAnxiety	Speilberger Trait Anxiety
STAI_sAnxiety	Speilberger State Anxiety
POMS_TotMdDisturb	Total Mood Disturbance
Barratt_tot	Barratt Impulsivity
MindWandering_Freq	Mind Wandering Frequency
NEO_N	NEO – Neuroticism
NEO_E	NEO – Extraversion
NEO_O	NEO – Openness to Experience
NEO_A	NEO – Agreeableness
NEO_C	NEO – Conscientiousness
TCI_Novelty	Novelty Seeking
TCI_RewardDependance	Reward Dependence
TCI_HarmAvoidance	Harm Avoidance
DOSPERT_taking	Risk Raking
DOSPERT_perception	Risk Perception
BISBAS_BAS_Drive	Behavioral Activation – Drive
BISBAS_BAS_Fun	Behavioral Activation – Fun
BISBAS_BAS_Reward	Behavioral Activation – Reward
BISBAS_BIS	Behavioral Inhibition

Table S4. Dictionary of 23 phenotypes in the GSP dataset.

HBN field	Description
SDQ_Conduct_Problems	SDQ: Conduct Problems Scale
SDQ_Difficulties_Total	SDQ: Total Difficulties Score
SDQ_Emotional_Problems	SDQ: Emotional Problems Scale
SDQ_Externalizing	SDQ: Externalizing Score
SDQ_Generating_Impact	SDQ: Generating Impact Scores
SDQ_Hyperactivity	SDQ: Hyperactivity Scale
SDQ_Internalizing	SDQ: Internalizing Score
SDQ_Peer_Problems	SDQ: Peer Problems Scale
SDQ_Prosocial	SDQ: Prosocial Scale
SRS_AWR_T	SRS: Social Awareness T-Score
SRS_COG_T	SRS: Social Cognition T-Score
SRS_COM_T	SRS: Social Communication T-Score
SRS_DSMRRB_T	SRS: Restricted Interests and Repetitive Behavior T-Score
SRS_MOT_T	SRS: Social Motivation T-Score
SRS_RRB_T	SRS: Restricted Interests and Repetitive Behavior T-Score
SRS_SCI_T	SRS: Social Communication and Interaction T-Score
SCQ_Total	Social Communication Questionnaire Total Score
ASSQ_Total	Autism Spectrum Screening Questionnaire Total Score
SWAN_IN_Avg	SWAN Rating Scale for ADHD: Inattention Average
SWAN_HY_Avg	SWAN Rating Scale for ADHD: Hyperactivity Average
ARI_S_Total_Score	Affective Reactivity Index Self-Report Total Score
ARI_P_Total_Score	Affective Reactivity Index Parent-Report Total Score
CBCL_AD_T	CBCL: Anxious/Depressed T Score
CBCL_WD_T	CBCL: Withdrawn/Depressed T Score
CBCL_SC_T	CBCL: Somatic Complaints T Score
CBCL_SP_T	CBCL: Social Problems T Score
CBCL_TP_T	CBCL: Thought Problems T Score
CBCL_AP_T	CBCL: Attention Problems T Score
CBCL_RBB_T	CBCL: Rule Breaking Behavior T Score
CBCL_AB_T	CBCL: Aggressive Behavior T Score
CBCL_OP	CBCL: Other Problems Raw Score
CBCL_Int_T	CBCL: Internalizing T Score
CBCL_Ext_T	CBCL: Externalizing T Score
CELF_Total	Clinical Evaluation of Language Fundamentals Total Score
WIAT_Num_Stnd	WIAT: Numerical Operations Standard Score

WIAT_Pseudo_Stnd	WIAT: Pseudo-word Decoding Standard Score
WIAT_Spell_Stnd	WIAT: Spelling Standard Score
WIAT_Word_Stnd	WIAT: Word Reading Standard Score
NIH7_Card	Dimensional Change Card Sort Age 3+ Age-adjusted Scale Score
NIH7_Flanker	Flanker Inhibitory Control and Attention Age 3+ Age-adjusted Score
NIH7_List	List Sorting Working Memory Age 7+ Age-adjusted Score
NIH7_Pattern	Pattern Comparison Process Speed 7+ Age-adjusted Score

Table S5. Dictionary of 42 phenotypes in the HBN dataset.

eNKI-RS field	Description
ANT_01	Alert Effect
ANT_02	Orienting Effect
ANT_03	Conflict Effect
DF_23	Total Set Loss Designs Scaled
DF_25	Total Repeated Designs Scaled
DF_27	Total Attempted Designs Scaled
DF_29	Percent Design Accuracy Scaled Score
DKEFSTMT_18	Visual Scanning Scaled
DKEFSTMT_19	Number Sequencing Scaled
DKEFSTMT_20	Letter Sequencing Scaled
DKEFSTMT_21	Number-Letter Switching Scaled
DKEFSTMT_22	Motor Speed Scaled
DKEFSTMT_24	Combined Number + Letter Sequencing Composite
DKEFSTMT_26	Switching vs Visual Scanning Scaled
DKEFSTMT_28	Switching Vs Number Sequencing Scaled
DKEFSTMT_30	Switching Vs Letter Sequencing Scaled
DKEFSTMT_32	Switching vs combined Number + letter sequencing Scaled
DKEFSTMT_34	Switching Vs Motor Speed Scaled
TOWER_47	Total Achievement Score Scaled
TOWER_49	Total Rule Violation Scaled
TOWER_51	Mean First Move Time Scaled
TOWER_53	Time Per Move Ratio Scaled
TOWER_55	Rule Violations Per Item Ratio Scaled
TOWER_57	Move Accuracy Ratio Scaled
DEHQ_16	Laterality Index
PTSDCH_55	PTSD Severity: Overall Score

INT_12	VCI Composite Score
INT_13	PRI Composite Score
INT_14	FSIQ - 4 Composite Score
WIAT_04	Word Reading Subtest Standard Score
WIAT_05	Numerical Operations Subtest Standard Score
WIAT_06	Spelling Subtest Standard Score
WIAT_08	Composite Standard Score
PENNCNP_0131	ER40 Correct Responses Median Response Time
PENNCNP_0136	ER40 Correct Anger Identifications
PENNCNP_0137	ER40 Correct Fear Identifications
PENNCNP_0139	ER40 Correct Happy Identifications
PENNCNP_0140	ER40 Correct No Emotion Identifications
VF_32	Category Fluency Scaled
VF_34	Category Switching Scaled
VF_35	Category Switching: Total Switching Accuracy
VF_37	Letter Fluency Vs Category Fluency Scaled
VF_39	Category Switching Vs Category Fluency Scaled
VF_41	First Interval Total Correct Scaled
VF_43	Second Interval Correct Scaled
VF_45	Third Interval Correct Scaled
VF_47	Fourth Interval Correct Scaled
VF_49	Set Loss Errors Scaled
VF_51	Repetition Errors Scaled
VF_54	Percent Set Loss Errors Scaled
DKEFSCWI_13	Color Naming Scaled
DKEFSCWI_14	Word Reading Scaled
DKEFSCWI_15	Inhibition Scaled
DKEFSCWI_16	Inhibition/Switching Scaled
DKEFSCWI_18	Combined Naming + Reading Comp Scaled
DKEFSCWI_20	Inhibition vs color naming Scaled
DKEFSCWI_22	Inhibition Vs Combined Naming + reading Scaled
DKEFSCWI_24	Inhibition Switching Vs Inhibition Scaled
DKEFSCWI_26	Inhibition/Switching Vs Color Naming Scaled
DKEFSCWI_28	Inhibition/Switching Vs Word Reading Scaled
DKEFSCWI_40	Total Errors Inhibition/Switching Scaled Score

Table S6. Dictionary of 61 phenotypes in the eNKI-RS dataset.

HCP-YA field	Description
PicSeq_Unadj	Visual Episodic Memory
CardSort_Unadj	Cognitive Flexibility (DCCS)
Flanker_Unadj	Inhibition (Flanker Task)
PMAT24_A_CR	Fluid Intelligence (PMAT)
ReadEng_Unadj	Vocabulary (Pronunciation)
PicVocab_Unadj	Vocabulary (Picture Matching)
ProcSpeed_Unadj	Processing Speed
DDic_AUC_40K	Delay Discounting
VSPLOT_TC	Spatial Orientation
SCPT_SPEC	Sustained Attention – Spec.
ListSort_Unadj	Working Memory (List Sorting)
MMSE_Score	Cognitive Status (MMSE)
PSQI_Score	Sleep Quality (PSQI)
Endurance_Unadj	Walking Endurance
GaitSpeed_Unadj	Walking Speed
Dexterity_Unadj	Manual Dexterity
Strength_Unadj	Grip Strength
Taste_Unadj	Taste Intensity
Emotion_Task_Face_Acc	Emotional Face Matching
Language_Task_Math_Avg_Difficulty_Level	Arithmetic
Language_Task_Story_Avg_Difficulty_Level	Story Comprehension
Relational_Task_Acc	Relational Processing
WM_Task_Acc	Working Memory (N-back)
NEOFAC_A	Agreeableness (NEO)
NEOFAC_O	Openness (NEO)
NEOFAC_C	Conscientiousness (NEO)
NEOFAC_E	Extraversion (NEO)
AngAggr_Unadj	Anger – Aggression
FearAffect_Unadj	Fear – Affect
Sadness_Unadj	Sadness
LifeSatisf_Unadj	Life Satisfaction
MeanPurp_Unadj	Meaning & Purpose
Loneliness_Unadj	Loneliness
PercStress_Unadj	Perceived Stress
SelfEff_Unadj	Self-Efficacy

Table S7. Dictionary of 35 phenotypes in the HCP-YA dataset.

HCP-Aging field	Description
LifeSatisf	Life Satisfaction
MeanPurp	Meaning & Purpose
PosAffect	Positive Affect
Sadness	Sadness
FearAffect	Fear – Affect
FearSomat	Fear – Somatic
AngAffect	Anger – Affect
AngHostil	Anger – Hostility
AngAggr	Anger – Aggression
ER40ANG	ER40 Correct Anger Identifications
ER40FEAR	ER40 Correct Fear Identifications
ER40NOE	ER40 Correct No Emotion Identifications
ER40SAD	ER40 Correct Sad Identifications
EmotSupp	Emotion Support
InstruSupp	Instrumental Support
Friendship	Friendship
Loneliness	Loneliness
PercReject	Perceived Rejection
PercHostil	Perceived Hostility
PercStress	Perceived Stress
SelfEff	Self-Efficacy
NEOFAC_A	Agreeableness (NEO)
NEOFAC_O	Openness (NEO)
NEOFAC_C	Conscientiousness (NEO)
NEOFAC_N	Neuroticism (NEO)
NEOFAC_E	Extraversion (NEO)
Endurance	Walking Endurance
GaitSpeed	Walking Speed
Strength	Grip Strength
PicSeq	Visual Episodic Memory
CardSort	Cognitive Flexibility (DCCS)
Flanker	Inhibition (Flanker Task)
ReadEng	Vocabulary (Pronunciation)
PicVocab	Vocabulary (Picture Matching)
ProcSpeed	Processing Speed
DDisc_AUC_40K	Delay Discounting

ListSort	Working Memory (List Sorting)
raw_vat	Raw Score for the Visual Acuity
raw_pain	Raw Score for the Pain Interference
winright_ncorr	Words in Noise Number Correct Right
psqi_total	Sleep Quality (PSQI)
tmta_raw	Trailmaking test part A: Time to completion
tmtb_raw	Trailmaking test Part B: Time to completion
pea_ravlt_sd_tc	RAVLT Short Delay Total Correct
moca_total	Montreal Cognitive Assessment Total Score

Table S8. Dictionary of 45 phenotypes in the HCP-Aging dataset.

S3. Bootstrapping procedure

Following our previous study (He et al., 2022), performance differences between algorithms were evaluated using a bootstrapping approach. More specifically, we performed bootstrap sampling of K-shot 1,000 times, i.e., we randomly picked K participants repeatedly with replacements from the meta-test dataset (so there might be duplicated participants). For each of the 1,000 bootstrapped samples, we applied all 5 algorithms (classical KRR, transfer learning, meta-matching with stacking, meta-matching with dataset stacking, and multilayer meta-matching), yielding prediction accuracies (Pearson's correlation and COD) in the remaining participants in the meta-test dataset. Note that the remaining participants might be more than N – K participants because some of the K participants might be duplicated (because of sampling with replacement).

Therefore, for each algorithm, we had 1,000 bootstrapped Pearson's correlation and 1,000 bootstrapped COD values. To explain the statistical testing procedure, we focus on COD in the following explanation for clarity, since the procedure for Pearson's correlation was the same as that for COD.

To compute the statistical difference between algorithm A and algorithm B, we calculated the difference between 1,000 bootstrapped samples for COD from algorithm A and 1,000 bootstrapped samples for COD from algorithm B, and we fitted a Gaussian distribution to the difference of 1,000 COD between A and B. We denoted the mean, standard deviation, and cumulative distribution function of the Gaussian distribution as μ, σ, and $CDF_{A\text{-}B}$, respectively. We then computed p-value by comparing 0 with $CDF_{A\text{-}B}$, i.e.:

$$p = \begin{cases} 2 \times \mathrm{CDF}_{\mathrm{A-B}}(0) & ,\text{if } \mu \geq 0 \\ 2 \times \left(1 - \mathrm{CDF}_{\mathrm{A-B}}(0)\right) & ,\text{if } \mu < 0 \end{cases}$$

P values were computed between all pairs of algorithms. Since there were multiple comparisons for different values of K (K = 10, 20, 50, 100, 200), different accuracy metrics (Pearson's correlation and COD), and for different pairs of algorithms, multiple comparisons were corrected using a false discovery rate (FDR) of $q < 0.05$. FDR was applied to all K-shots and across all pairs of algorithms and both evaluation metrics.

Supplementary Results

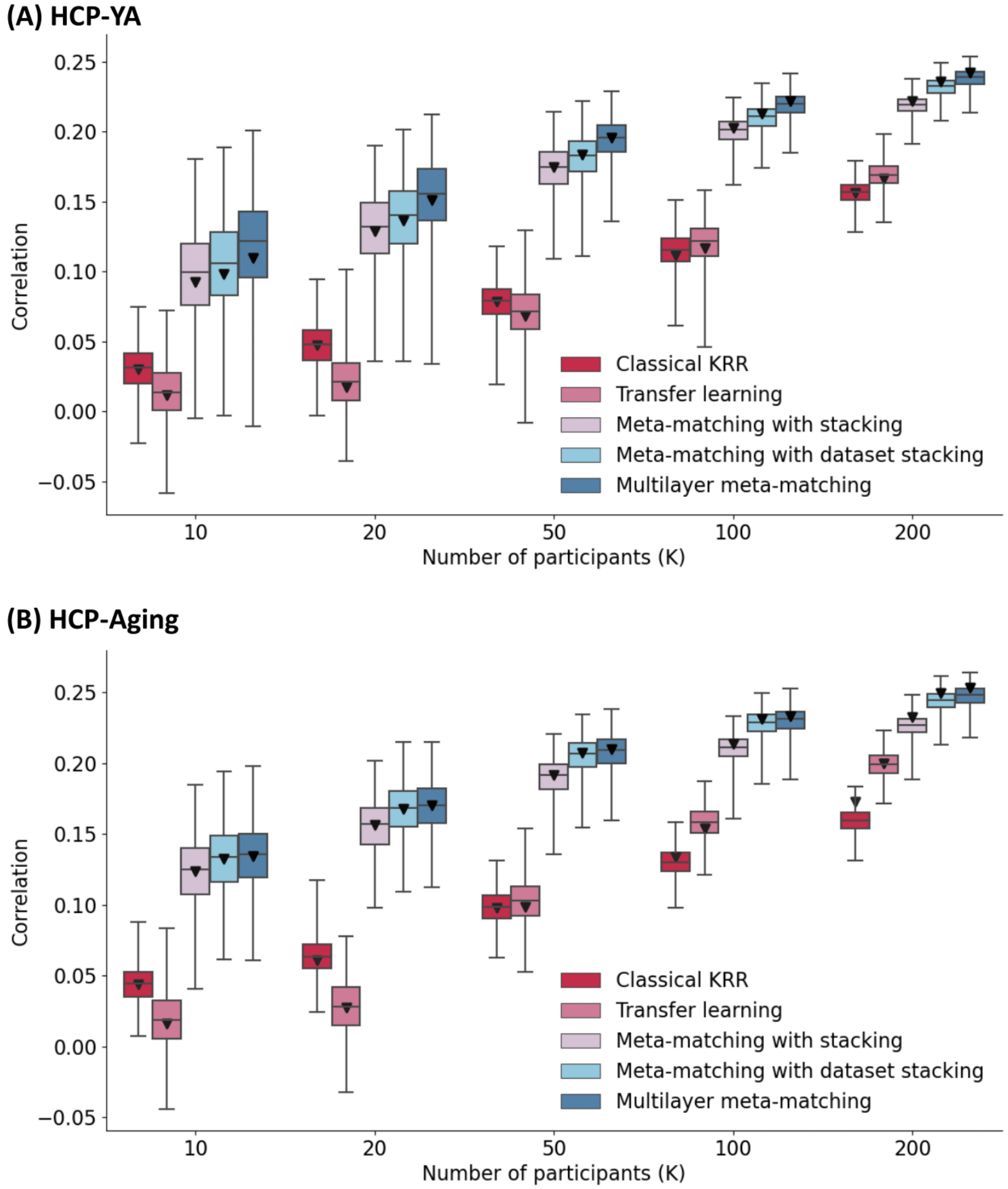

Figure S1. Multilayer meta-matching outperformed meta-matching with stacking on HCP-YA bootstrap experiment. (A) Phenotypic prediction performance in terms of Pearson's correlation (averaged across 35 meta-test phenotypes) on HCP-YA bootstrap samples. Horizontal axis is the number of few-shot participants (K); Vertical axis is Pearson's correlation of phenotypic prediction. Boxplots represent variability across 1,000 bootstrapped K-shot, and different colors stand for different approaches. Inverted triangles represent the average prediction performance (correlation) on 100 random K-shot. (B) Prediction performance in terms of COD on HCP-YA bootstrap samples. All settings were the same as (A) but used COD as the metric.

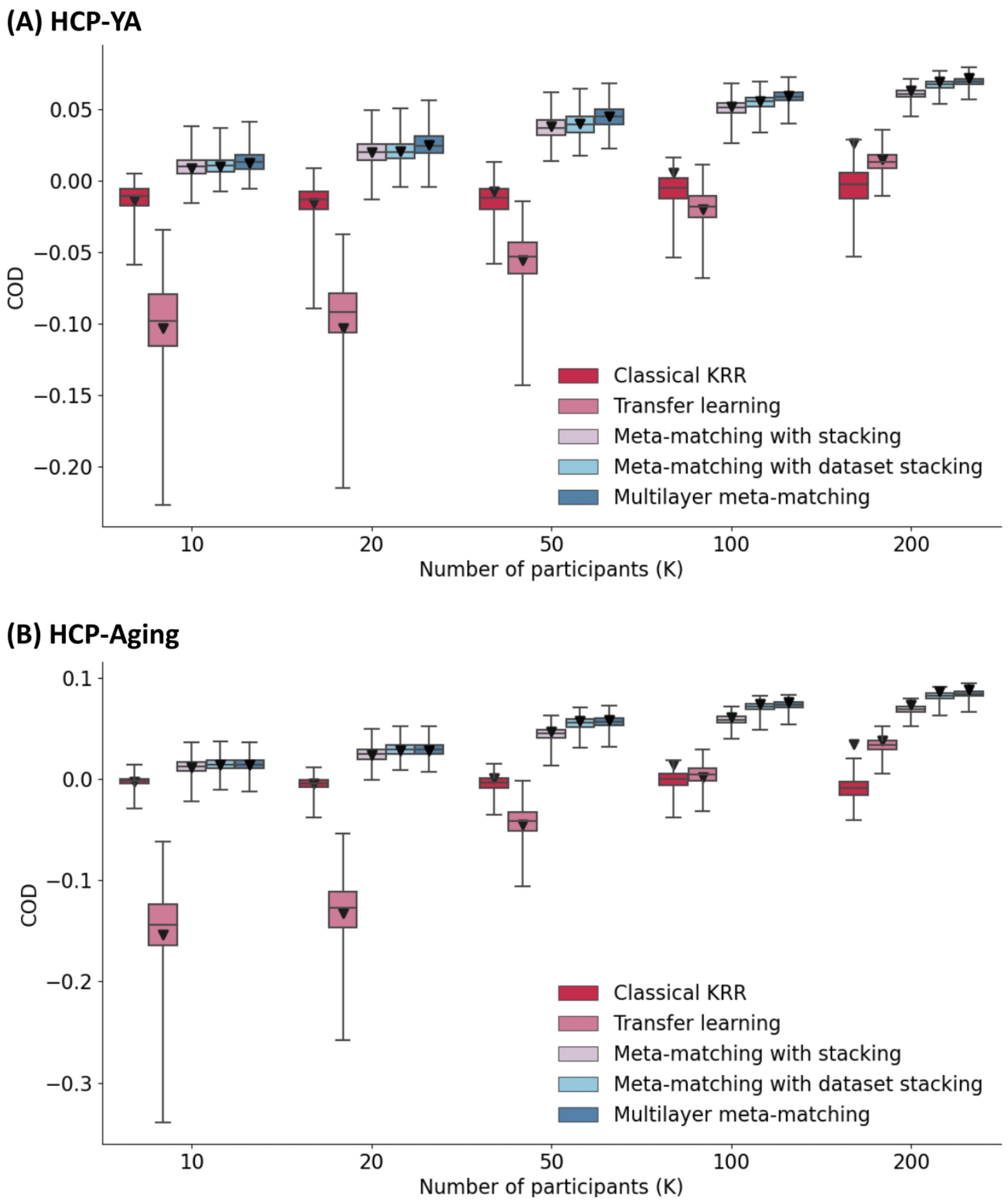

Figure S2. Multilayer meta-matching outperformed meta-matching with stacking on HCP-Aging bootstrap experiment. (A) Phenotypic prediction performance in terms of Pearson's correlation (averaged across 45 meta-test phenotypes) on HCP-Aging bootstrap samples. Horizontal axis is the number of participants (K); Vertical axis is Pearson's correlation of phenotypic prediction. Boxplots represent variability across 1,000 bootstrapped K-shot, and different colors stand for different algorithms. Inverted triangles represent the average prediction performance (correlation) on 100 random K-shot. (B) Prediction performance in terms of COD on HCP-Aging bootstrap samples. All settings were the same as A but used COD as the metric.

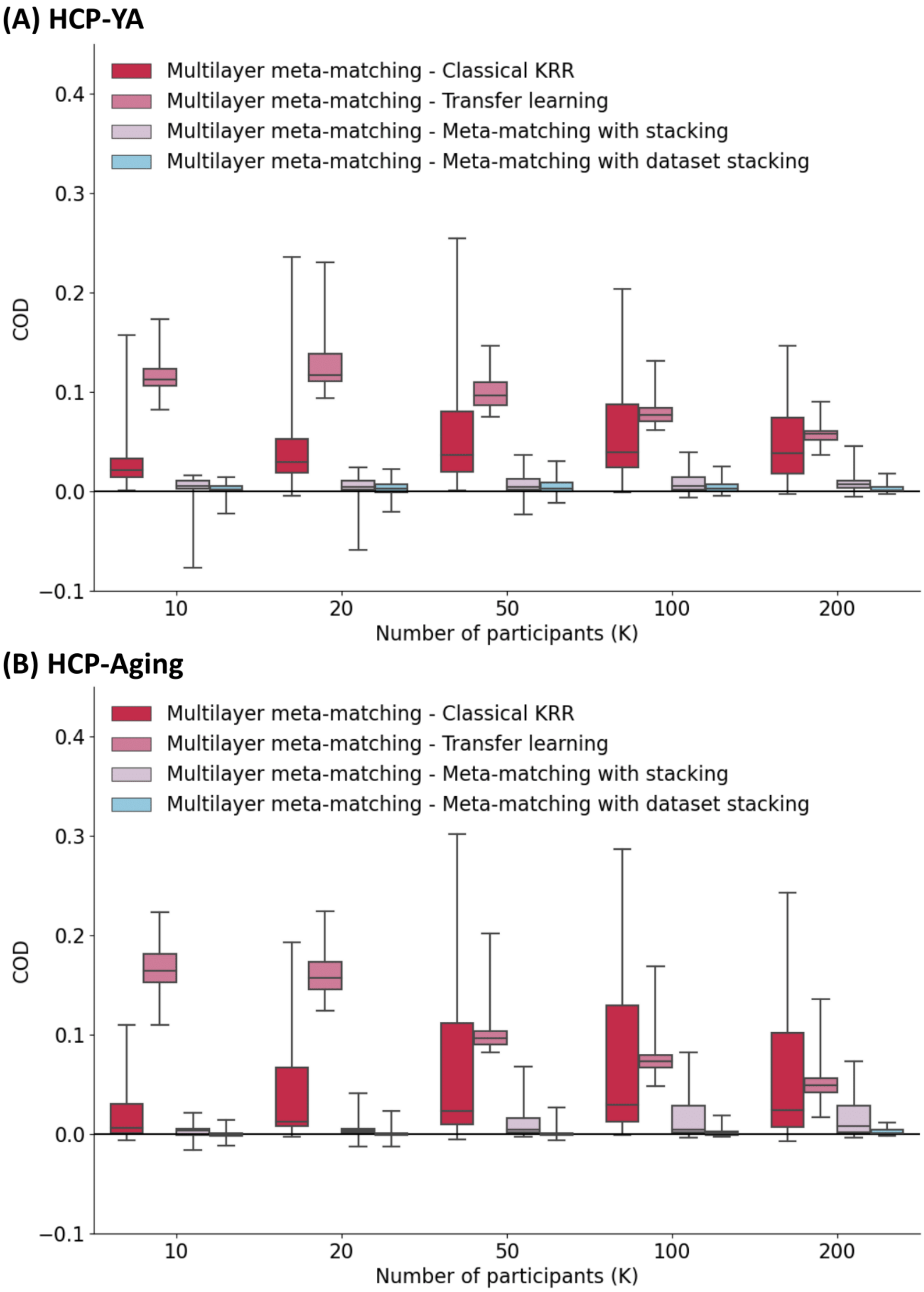

Figure S3. Numerical improvement in prediction performance (COD) across different phenotypes in the HCP-YA and HCP-Aging datasets. (A) Phenotypic prediction performance (averaged across 100 repetitions of sampling K participants) in the HCP-YA dataset. Horizontal axis is the number of participants in the HCP-YA dataset used to adapt the models trained from the meta-training datasets. Boxplots represent variability across the 35 HCP-YA phenotypes. The bottom and top edges of the box indicate the 25th and 75th percentiles, respectively. Whiskers correspond to 1.5 times the interquartile range. (B) Same plot as panel A except that the analyses were performed in the HCP-Aging dataset with 45 phenotypes.

**Correlation**		Classical KRR	Transfer learning	MM w/ stacking	MM w/ dataset stacking	Multilayer MM
	Classical KRR	-	0.494<br>0.265<br>0.705<br>0.732<br>0.225	**0.0301**<br>**0.00191**<br>**1.71e-07**<br>**4.71e-10**<br>**5.24e-10**	**0.0131**<br>**2.77**<br>**3.31e-10**<br>**6.79e-14**<br>**3.33e-16**	**0.00738**<br>**2.68e-05**<br>**4.95e-13**<br>**1.11e-15**<br>**0.0**
	Transfer learning	0.494<br>0.265<br>0.705<br>0.732<br>0.225	-	**0.0190**<br>**4.39e-04**<br>**2.56e-07**<br>**1.02e-07**<br>**4.94e-07**	**0.0121**<br>**1.41e-04**<br>**1.14e-08**<br>**8.87e-10**<br>**5.30e-11**	**0.00770**<br>**2.21e-05**<br>**1.55e-10**<br>**7.43e-11**<br>**2.24e-12**
	MM w/ stacking	**0.0301**<br>**0.00191**<br>**1.71e-07**<br>**4.71e-10**<br>**5.24e-10**	**0.0190**<br>**4.39e-04**<br>**2.56e-07**<br>**1.02e-07**<br>**4.94e-07**	-	0.448<br>0.302<br>0.108<br>**0.0124**<br>**9.25e-05**	0.174<br>0.0429<br>**0.00432**<br>**4.61e-04**<br>**1.27**
	MM w/ dataset stacking	**0.0131**<br>**2.77**<br>**3.31e-10**<br>**6.79e-14**<br>**3.33e-16**	**0.0121**<br>**1.41e-04**<br>**1.14e-08**<br>**8.87e-10**<br>**5.30e-11**	0.448<br>0.302<br>0.108<br>**0.0124**<br>**9.25e-05**	-	0.164<br>**0.0304**<br>**0.00615**<br>**0.00410**<br>**0.00297**
	Multilayer MM	**0.00738**<br>**2.68e-05**<br>**4.95e-13**<br>**1.11e-15**<br>**0.0**	**0.00770**<br>**2.21e-05**<br>**1.55e-10**<br>**7.43e-11**<br>**2.24e-12**	0.174<br>0.0429<br>**0.00432**<br>**4.61e-04**<br>**1.27**	0.164<br>**0.0304**<br>**0.00615**<br>**0.00410**<br>**0.00297**	-

**COD**		Classical KRR	Transfer learning	MM w/ stacking	MM w/ dataset stacking	Multilayer MM
	Classical KRR	-	**0.00200**<br>**9.80e-04**<br>**0.0428**<br>**0.414**<br>**0.222**	0.0797<br>**0.00821**<br>**1.01e-04**<br>**8.00e-07**<br>**1.09e-06**	0.0460<br>**0.00481**<br>**3.29e-05**<br>**6.00e-08**<br>**6.71e-08**	**0.0351**<br>**0.00278**<br>**3.95e-06**<br>**5.92e-09**<br>**2.25e-08**
	Transfer learning	**0.00200**<br>**9.80e-04**<br>**0.0428**<br>**0.414**<br>**0.222**	-	**4.95e-05**<br>**6.14e-07**<br>**1.10e-08**<br>**8.29e-11**<br>**1.47e-12**	**3.65e-05**<br>**3.36e-07**<br>**2.15e-09**<br>**1.19e-12**<br>**2.22e-16**	**2.52e-05**<br>**2.26e-07**<br>**3.15e-10**<br>**2.04e-13**<br>**0.0**
	MM w/ stacking	0.0797<br>**0.00821**<br>**1.01e-04**<br>**8.00e-07**<br>**1.09e-06**	**4.95e-05**<br>**6.14e-07**<br>**1.10e-08**<br>**8.29e-11**<br>**1.47e-12**	-	0.773<br>0.760<br>0.393<br>0.0512<br>**2.01e-04**	0.425<br>0.219<br>**0.0226**<br>**0.00125**<br>**5.32e-06**
	MM w/ dataset stacking	0.0460<br>**0.00481**<br>**3.29e-05**<br>**6.00e-08**<br>**6.71e-08**	**3.65e-05**<br>**3.36e-07**<br>**2.15e-09**<br>**1.19e-12**<br>**2.22e-16**	0.773<br>0.760<br>0.393<br>0.0512<br>**2.01e-04**	-	0.336<br>0.110<br>**0.00498**<br>**0.00508**<br>**0.0202**
	Multilayer MM	**0.0351**<br>**0.00278**<br>**3.95e-06**<br>**5.92e-09**<br>**2.25e-08**	**2.52e-05**<br>**2.26e-07**<br>**3.15e-10**<br>**2.04e-13**<br>**0.0**	0.425<br>0.219<br>**0.0226**<br>**0.00125**<br>**5.32e-06**	0.336<br>0.110<br>**0.00498**<br>**0.00508**<br>**0.0202**	-

Table S9. Statistical differences in prediction accuracy in terms of Pearson's correlation (upper) and COD (bottom) between all pairs of approaches in the HCP-YA meta-test

dataset. Here 'MM' stands for 'meta-matching' and 'w/' is short for 'with'. Each cell contains five p values, corresponding to K = 10, 20, 50, 100 and 200 respectively. Bolded p values are statistically significant after FDR correction with q < 0.05.

		Classical KRR	Transfer learning	MM w/ stacking	MM w/ dataset stacking	Multilayer MM
**Correlation**	Classical KRR	-	0.260<br>0.105<br>0.813<br>**0.0353**<br>**2.06e-04**	**3.41e-04**<br>**8.02e-07**<br>**3.30e-09**<br>**1.03e-10**<br>**5.98e-10**	**3.60e-05**<br>**6.52e-09**<br>**2.25e-13**<br>**0.0**<br>**0.0**	**1.30e-05**<br>**7.94e-10**<br>**1.63e-14**<br>**0.0**<br>**0.0**
	Transfer learning	0.260<br>0.105<br>0.813<br>**0.0353**<br>**2.06e-04**	-	**2.58e-04**<br>**1.74e-07**<br>**2.27e-07**<br>**1.35e-05**<br>**0.00307**	**6.57e-05**<br>**1.18e-08**<br>**3.39e-10**<br>**2.27e-09**<br>**3.89e-07**	**3.73e-05**<br>**3.75e-09**<br>**8.25e-11**<br>**5.49e-10**<br>**5.99e-08**
	MM w/ stacking	**3.41e-04**<br>**8.02e-07**<br>**3.30e-09**<br>**1.03e-10**<br>**5.98e-10**	**2.58e-04**<br>**1.74e-07**<br>**2.27e-07**<br>**1.35e-05**<br>**0.00307**	-	0.0882<br>**0.00782**<br>**7.21e-06**<br>**2.22e-08**<br>**4.84e-08**	0.112<br>**0.0139**<br>**8.71e-05**<br>**3.69e-07**<br>**1.12e-08**
	MM w/ dataset stacking	**3.60e-05**<br>**6.52e-09**<br>**2.25e-13**<br>**0.0**<br>**0.0**	**6.57e-05**<br>**1.18e-08**<br>**3.39e-10**<br>**2.27e-09**<br>**3.89e-07**	0.0882<br>**0.00782**<br>**7.21e-06**<br>**2.22e-08**<br>**4.84e-08**	-	0.517<br>0.399<br>0.282<br>0.174<br>**0.0289**
	Multilayer MM	**1.30e-05**<br>**7.94e-10**<br>**1.63e-14**<br>**0.0**<br>**0.0**	**3.73e-05**<br>**3.75e-09**<br>**8.25e-11**<br>**5.49e-10**<br>**5.99e-08**	0.112<br>**0.0139**<br>**8.71e-05**<br>**3.69e-07**<br>**1.12e-08**	0.517<br>0.399<br>0.282<br>0.174<br>**0.0289**	-
		Classical KRR	Transfer learning	MM w/ stacking	MM w/ dataset stacking	Multilayer MM
**COD**	Classical KRR	-	**1.89e-05**<br>**4.43e-06**<br>**0.0150**<br>0.694<br>**4.41e-05**	0.0899<br>**0.00128**<br>**9.21e-09**<br>**3.56e-11**<br>**2.55e-15**	**0.0210**<br>**3.65e-05**<br>**7.68e-14**<br>**0.0**<br>**0.0**	**0.0210**<br>**3.17e-05**<br>**3.13e-14**<br>**0.0**<br>**0.0**
	Transfer learning	**1.89e-05**<br>**4.43e-06**<br>**0.0150**<br>0.694<br>**4.41e-05**	-	**2.30e-06**<br>**1.02e-08**<br>**4.50e-09**<br>**7.79e-09**<br>**3.86e-07**	**1.66e-06**<br>**4.60e-09**<br>**2.66e-11**<br>**2.18e-13**<br>**7.27e-13**	**1.70e-06**<br>**4.18e-09**<br>**1.261e-11**<br>**6.81e-14**<br>**1.24e-13**
	MM w/ stacking	0.0899<br>**0.00128**<br>**9.21e-09**<br>**3.56e-11**<br>**2.55e-15**	**2.30e-06**<br>**1.02e-08**<br>**4.50e-09**<br>**7.79e-09**<br>**3.86e-07**	-	0.426<br>0.142<br>**2.22e-04**<br>**6.50e-08**<br>**1.17e-09**	0.499<br>0.172<br>**2.17e-04**<br>**2.50e-08**<br>**1.10e-10**
	MM w/ dataset stacking	**0.0210**<br>**3.65e-05**<br>**7.68e-14**<br>**0.0**<br>**0.0**	**1.66e-06**<br>**4.60e-09**<br>**2.66e-11**<br>**2.18e-13**<br>**7.27e-13**	0.426<br>0.142<br>**2.22e-04**<br>**6.50e-08**<br>**1.17e-09**	-	0.997<br>0.862<br>0.348<br>0.0858<br>**0.00865**
	Multilayer MM	**0.0210**<br>**3.17e-05**<br>**3.13e-14**<br>**0.0**<br>**0.0**	**1.70e-06**<br>**4.18e-09**<br>**1.261e-11**<br>**6.81e-14**<br>**1.24e-13**	0.499<br>0.172<br>**2.17e-04**<br>**2.50e-08**<br>**1.10e-10**	0.997<br>0.862<br>0.348<br>0.0858<br>**0.00865**	-

Table S10. Statistical differences in prediction accuracy in terms of Pearson's correlation (upper) and COD (bottom) between all pairs of approaches in the HCP-Aging meta-test dataset. Here 'MM' stands for 'meta-matching', and 'w/' is short for 'with'. Each cell contains five p values, corresponding to K = 10, 20, 50, 100 and 200 respectively. Bolded p values are statistically significant after FDR correction with q < 0.05.

Datasets	K	Multilayer MM v.s. Classical KRR	Multilayer MM v.s. Transfer learning	Multilayer MM v.s. MM w/ stacking	Multilayer MM v.s. MM w/ dataset stacking
HCP-YA	10	100%	100%	85.7%	71.4%
	20	97.1%	100%	85.7%	62.8%
	50	100%	100%	85.7%	77.1%
	100	97.1%	100%	82.9%	71.4%
	200	97.1%	100%	85.7%	71.4%
HCP-Aging	10	77.8%	100%	73.3%	48.9%
	20	91.1%	100%	86.7%	44.4%
	50	95.6%	100%	91.1%	51.1%
	100	93.3%	100%	86.7%	62.2%
	200	88.9%	100%	84.4%	64.4%

Table S11. Percentages of phenotypes with numerical improvement in prediction performance (COD).

**HCP-YA phenotypes**	Multilayer MM v.s. Classical KRR	Multilayer MM v.s. Transfer learning	Multilayer MM v.s. MM w/ stacking	Multilayer MM v.s. MM w/ dataset stacking	Multilayer MM prediction correlation
Language_Task_Story_Avg_Difficulty_Level	2.06E-01	1.58E-01	1.94E-02	1.72E-02	3.88E-01
ReadEng_Unadj	2.05E-01	2.25E-01	5.49E-02	2.67E-02	4.23E-01
WM_Task_Acc	2.03E-01	1.26E-01	2.23E-02	8.70E-03	3.83E-01
PMAT24_A_CR	1.93E-01	1.61E-01	2.24E-02	1.36E-02	3.56E-01
VSPLOT_TC	1.92E-01	1.47E-01	1.39E-02	1.06E-02	3.56E-01
Strength_Unadj	1.89E-01	5.77E-02	2.28E-03	-1.65E-03	6.66E-01
Relational_Task_Acc	1.89E-01	1.64E-01	1.99E-02	1.32E-02	3.57E-01
ListSort_Unadj	1.86E-01	1.57E-01	2.96E-02	1.66E-02	3.01E-01
PicVocab_Unadj	1.86E-01	1.88E-01	4.02E-02	1.86E-02	4.04E-01
Endurance_Unadj	1.69E-01	1.01E-01	6.77E-03	-1.20E-03	3.81E-01
Dexterity_Unadj	1.55E-01	1.12E-01	-4.58E-03	-4.50E-03	2.21E-01
PicSeq_Unadj	1.55E-01	1.27E-01	7.30E-03	3.38E-04	2.64E-01
Taste_Unadj	1.48E-01	1.09E-01	-5.42E-03	-5.40E-03	1.87E-01
Language_Task_Math_Avg_Difficulty_Level	1.43E-01	1.40E-01	3.56E-02	2.74E-02	2.06E-01

CardSort_Unadj	1.41E-01	1.42E-01	4.55E-02	9.81E-03	2.44E-01
LifeSatisf_Unadj	1.29E-01	1.24E-01	1.87E-03	7.65E-03	1.83E-01
Flanker_Unadj	1.27E-01	8.53E-02	1.35E-02	3.78E-03	1.77E-01
AngAggr_Unadj	1.05E-01	1.10E-01	8.43E-04	-5.47E-03	2.78E-01
MMSE_Score	1.04E-01	9.55E-02	2.88E-02	1.98E-02	1.29E-01
DDisc_AUC_40K	9.70E-02	1.48E-01	3.69E-02	9.12E-03	2.64E-01
SCPT_SPEC	9.56E-02	1.07E-01	-3.53E-03	1.98E-03	1.70E-01
NEOFAC_A	9.29E-02	9.45E-02	-1.24E-02	-8.78E-03	2.34E-01
NEOFAC_O	8.98E-02	1.13E-01	5.55E-02	3.30E-02	1.73E-01
ProcSpeed_Unadj	8.08E-02	1.05E-01	3.32E-02	2.05E-02	1.45E-01
PSQI_Score	7.63E-02	9.39E-02	8.96E-03	1.11E-02	1.42E-01
PercStress_Unadj	5.72E-02	7.82E-02	1.93E-02	1.49E-02	1.11E-01
NEOFAC_C	5.08E-02	9.25E-02	3.11E-02	4.29E-03	1.37E-01
FearAffect_Unadj	4.44E-02	6.67E-02	2.05E-02	1.20E-02	9.54E-02
MeanPurp_Unadj	3.47E-02	4.83E-02	2.13E-02	2.06E-03	8.18E-02
SelfEff_Unadj	2.42E-02	4.61E-02	8.79E-03	-1.85E-03	7.90E-02
Sadness_Unadj	1.62E-02	4.40E-02	3.85E-02	1.97E-02	6.15E-02
Loneliness_Unadj	4.22E-03	2.34E-02	3.60E-02	1.80E-02	4.30E-02
NEOFAC_E	-2.04E-03	3.70E-02	8.15E-03	1.89E-03	4.69E-02
GaitSpeed_Comp	-1.05E-02	2.04E-02	-8.49E-04	-7.70E-03	3.16E-02
Emotion_Task_Face_Acc	-2.04E-02	3.16E-02	1.10E-02	6.70E-03	4.49E-02

Table S12. Numerical improvement in prediction performance (Pearson's correlation) of multilayer meta-matching on each HCP-YA phenotypes (K = 100). Rows were sorted by on the improvement of multilayer meta-matching over classical KRR (from largest to smallest). Negative values imply that multilayer meta-matching perform worse than baseline. The last column corresponds to the actual prediction accuracy of multilayer meta-matching.

**HCP-YA phenotypes**	Multilayer MM v.s. Classical KRR	Multilayer MM v.s. Transfer learning	Multilayer MM v.s. MM w/ stacking	Multilayer MM v.s. MM w/ dataset stacking	Multilayer MM prediction COD
Strength_Unadj	2.00E-01	1.09E-01	-3.04E-02	-1.32E-02	3.36E-01
Language_Task_Story_Avg_Difficulty_Level	9.09E-02	1.01E-01	1.87E-02	1.37E-02	9.85E-02
ReadEng_Unadj	8.61E-02	1.17E-01	3.27E-02	1.97E-02	1.06E-01
WM_Task_Acc	8.58E-02	9.06E-02	1.69E-02	1.06E-02	9.49E-02
PMAT24_A_CR	7.92E-02	8.21E-02	1.17E-02	8.49E-03	7.88E-02
PicVocab_Unadj	7.88E-02	1.14E-01	2.50E-02	1.40E-02	1.02E-01
Relational_Task_Acc	7.62E-02	1.02E-01	1.58E-02	1.07E-02	8.22E-02

Endurance_Unadj	7.52E-02	8.84E-02	1.68E-03	3.18E-04	9.36E-02
VSPLOT_TC	7.30E-02	8.75E-02	9.22E-03	6.91E-03	7.80E-02
ListSort_Unadj	5.93E-02	7.85E-02	9.31E-03	5.87E-03	5.15E-02
PicSeq_Unadj	4.81E-02	7.22E-02	3.06E-03	1.22E-03	3.63E-02
AngAggr_Unadj	4.30E-02	8.24E-02	-3.17E-03	-3.41E-03	4.75E-02
CardSort_Unadj	4.30E-02	9.08E-02	1.54E-02	5.53E-03	3.52E-02
Dexterity_Unadj	4.13E-02	7.30E-02	-1.74E-03	-1.59E-03	2.98E-02
DDic_AUC_40K	3.82E-02	9.67E-02	1.36E-02	3.88E-03	3.94E-02
Language_Task_Math_Avg_Difficulty_Level	3.80E-02	8.19E-02	1.19E-02	7.37E-03	2.34E-02
Taste_Unadj	3.22E-02	7.41E-02	-8.27E-04	-9.26E-04	1.99E-02
NEOFAC_O	3.11E-02	7.35E-02	1.35E-02	6.77E-03	1.70E-02
LifeSatisf_Unadj	3.09E-02	7.48E-02	2.84E-03	2.29E-03	1.95E-02
NEOFAC_A	3.08E-02	7.50E-02	-5.91E-03	-2.95E-03	3.26E-02
Flanker_Unadj	3.04E-02	6.67E-02	5.02E-03	1.62E-03	1.77E-02
MMSE_Score	2.55E-02	7.29E-02	6.56E-03	2.69E-03	8.74E-03
SCPT_SPEC	2.36E-02	7.40E-02	5.45E-04	2.48E-04	1.71E-02
PSQI_Score	2.17E-02	8.37E-02	3.86E-03	1.81E-03	1.10E-02
ProcSpeed_Unadj	2.09E-02	7.78E-02	7.63E-03	4.71E-03	1.13E-02
NEOFAC_C	1.88E-02	7.42E-02	3.30E-03	-2.19E-04	8.13E-03
FearAffect_Unadj	1.80E-02	7.07E-02	4.18E-03	1.61E-03	4.44E-03
SelfEff_Unadj	1.69E-02	7.00E-02	2.66E-03	-4.77E-04	2.88E-03
PercStress_Unadj	1.56E-02	7.29E-02	6.03E-03	2.77E-03	6.68E-03
GaitSpeed_Unadj	1.32E-02	6.61E-02	1.93E-03	-1.41E-03	-4.94E-03
MeanPurp_Unadj	1.23E-02	7.73E-02	3.71E-03	-5.78E-05	3.92E-04
Sadness_Unadj	1.20E-02	7.51E-02	6.48E-03	1.83E-03	-1.79E-04
NEOFAC_E	1.16E-02	8.24E-02	3.63E-03	-1.44E-04	-3.34E-03
Loneliness_Unadj	9.83E-03	6.98E-02	6.25E-03	1.39E-03	-4.39E-03
Emotion_Task_Face_Acc	-1.42E-03	6.70E-02	4.44E-03	2.86E-04	-3.64E-03

Table S13. Numerical improvement in prediction performance (COD) of multilayer meta-matching on each HCP-YA phenotypes (K = 100). Rows were sorted by on the improvement of multilayer meta-matching over classical KRR (from largest to smallest). Negative values imply that multilayer meta-matching perform worse than baseline. The last column corresponds to the actual prediction accuracy of multilayer meta-matching.

**HCP-Aging phenotypes**	Multilayer MM v.s. Classical KRR	Multilayer MM v.s. Transfer learning	Multilayer MM v.s. MM w/ stacking	Multilayer MM v.s. MM w/ dataset stacking	Multilayer MM prediction correlation

Strength	3.02E-01	5.35E-02	2.13E-02	-6.73E-05	6.37E-01
moca_total	2.43E-01	1.23E-01	4.59E-02	6.13E-03	4.14E-01
PicVocab	2.37E-01	2.31E-01	4.33E-02	1.01E-02	5.36E-01
CardSort	2.21E-01	1.31E-01	2.94E-02	3.49E-03	4.21E-01
ListSort	2.08E-01	1.16E-01	3.69E-02	4.02E-03	4.40E-01
Flanker	2.04E-01	8.81E-02	4.28E-02	7.25E-03	4.66E-01
ReadEng	2.03E-01	1.86E-01	9.27E-02	1.57E-02	4.30E-01
Endurance	2.03E-01	9.04E-02	4.10E-02	2.31E-03	5.38E-01
pea_ravlt_sd_tc	1.96E-01	8.07E-02	3.47E-02	1.74E-03	4.87E-01
ProcSpeed	1.86E-01	1.13E-01	2.25E-02	2.72E-03	4.88E-01
PicSeq	1.83E-01	1.04E-01	2.74E-02	-3.29E-03	4.29E-01
tmtb_raw	1.79E-01	9.01E-02	3.94E-02	7.26E-03	3.60E-01
winright_ncorr	1.73E-01	8.46E-02	9.00E-03	-1.51E-03	4.73E-01
tmta_raw	1.69E-01	7.16E-02	3.24E-02	7.36E-03	4.12E-01
raw_vat	1.67E-01	7.88E-02	2.25E-02	3.85E-03	4.11E-01
DDisc_AUC_40K	1.41E-01	1.16E-01	5.08E-02	8.22E-03	2.14E-01
ER40ANG	1.28E-01	7.55E-02	2.27E-02	1.78E-03	2.70E-01
GaitSpeed	1.25E-01	8.86E-02	3.15E-02	4.20E-03	2.18E-01
AngAffect	9.84E-02	1.09E-01	-2.55E-03	-3.53E-03	1.44E-01
NEOFAC_O	9.46E-02	1.54E-01	7.38E-02	1.47E-02	2.47E-01
AngHostil	9.22E-02	9.75E-02	7.31E-03	-4.65E-04	1.96E-01
ER40SAD	9.09E-02	9.49E-02	-1.13E-02	-6.02E-03	2.02E-01
LifeSatisf	8.89E-02	1.03E-01	1.95E-02	3.42E-03	2.12E-01
AngAggr	7.73E-02	5.82E-02	2.93E-03	-6.35E-03	1.14E-01
Loneliness	7.15E-02	9.81E-02	-7.63E-04	-1.18E-03	1.65E-01
PercHostil	6.48E-02	5.93E-02	1.72E-02	3.86E-03	1.05E-01
NEOFAC_A	5.57E-02	7.59E-02	1.16E-02	3.13E-03	1.25E-01
InstruSupp	5.45E-02	8.76E-02	1.05E-02	1.66E-03	1.30E-01
FearAffect	5.26E-02	5.63E-02	-9.10E-03	-7.12E-03	1.23E-01
ER40NOE	5.18E-02	4.43E-02	-1.77E-03	-6.83E-03	6.19E-02
Friendship	4.91E-02	4.43E-02	-1.42E-02	-4.21E-03	1.34E-01
PercStress	4.59E-02	7.01E-02	-4.43E-04	-3.17E-03	1.16E-01
csq_psqi_se	4.36E-02	3.26E-02	5.14E-03	-2.34E-04	3.01E-02
NEOFAC_N	4.01E-02	7.77E-02	-6.52E-03	-5.55E-03	1.42E-01
raw_pain	2.76E-02	3.64E-02	9.26E-03	-1.04E-03	7.30E-02
Sadness	2.48E-02	7.97E-02	6.62E-03	-2.87E-03	1.11E-01
PosAffect	2.26E-02	4.44E-02	8.88E-03	3.58E-03	6.52E-02
ER40FEAR	1.79E-02	6.01E-02	1.25E-02	-6.56E-04	1.23E-01
FearSomat	1.61E-02	3.56E-02	6.17E-03	5.88E-03	4.27E-02

NEOFAC_E	1.42E-03	7.56E-02	3.45E-02	1.83E-02	9.37E-02
NEOFAC_C	-6.33E-03	4.16E-02	3.20E-02	8.91E-03	5.77E-02
MeanPurp	-2.66E-02	2.96E-02	1.10E-02	3.86E-03	4.16E-02
PercReject	-2.83E-02	-1.44E-02	-4.64E-03	-2.69E-03	-3.49E-03
SelfEff	-4.52E-02	-1.92E-02	-3.37E-03	-4.80E-05	-1.98E-02
EmotSupp	-5.53E-02	8.31E-03	1.43E-02	7.90E-03	2.82E-02

Table S14. Numerical improvement in prediction performance (Pearson's correlation) of multilayer meta-matching on each HCP-Aging phenotypes (K = 100). Rows were sorted by on the improvement of multilayer meta-matching over classical KRR (from largest to smallest). Negative values imply that multilayer meta-matching perform worse than baseline. The last column corresponds to the actual prediction accuracy of multilayer meta-matching.

**HCP-Aging phenotypes**	Multilayer MM v.s. Classical KRR	Multilayer MM v.s. Transfer learning	Multilayer MM v.s. MM w/ stacking	Multilayer MM v.s. MM w/ dataset stacking	Multilayer MM prediction COD
Strength	2.86E-01	5.04E-02	2.40E-02	-2.16E-03	3.83E-01
PicVocab	1.85E-01	1.69E-01	6.61E-02	1.91E-02	2.61E-01
Endurance	1.68E-01	7.79E-02	4.77E-02	1.87E-03	2.69E-01
pea_ravlt_sd_tc	1.56E-01	6.33E-02	3.52E-02	1.15E-03	2.22E-01
Flanker	1.51E-01	6.10E-02	4.16E-02	7.17E-03	2.00E-01
ProcSpeed	1.46E-01	8.59E-02	2.47E-02	2.32E-03	2.23E-01
moca_total	1.45E-01	8.62E-02	4.28E-02	6.02E-03	1.60E-01
ListSort	1.36E-01	7.81E-02	3.79E-02	3.27E-03	1.74E-01
CardSort	1.35E-01	8.67E-02	2.90E-02	3.38E-03	1.61E-01
winright_ncorr	1.33E-01	6.49E-02	6.77E-03	-2.67E-03	2.05E-01
PicSeq	1.32E-01	7.29E-02	2.90E-02	-1.87E-03	1.71E-01
ReadEng	1.29E-01	1.11E-01	8.24E-02	1.64E-02	1.63E-01
raw_vat	1.13E-01	5.51E-02	1.95E-02	3.01E-03	1.56E-01
tmta_raw	1.13E-01	4.88E-02	3.11E-02	7.09E-03	1.54E-01
tmtb_raw	8.57E-02	8.02E-02	3.50E-02	4.76E-03	1.12E-01
ER40ANG	5.83E-02	4.85E-02	1.12E-02	1.18E-03	6.21E-02
DDisc_AUC_40K	4.85E-02	7.18E-02	2.10E-02	3.27E-03	3.82E-02
NEOFAC_O	4.54E-02	8.41E-02	3.02E-02	5.76E-03	5.17E-02
GaitSpeed	3.99E-02	5.48E-02	9.51E-03	1.21E-03	3.78E-02
LifeSatisf	3.57E-02	6.68E-02	7.48E-03	1.09E-03	3.71E-02
ER40SAD	3.43E-02	7.24E-02	-3.54E-03	-1.98E-03	3.38E-02
AngHostil	3.35E-02	6.90E-02	5.56E-04	-4.96E-04	3.21E-02

Loneliness	2.91E-02	7.46E-02	3.33E-05	-6.00E-04	2.09E-02
AngAffect	2.72E-02	8.50E-02	-5.20E-04	-1.28E-03	1.50E-02
AngAggr	2.50E-02	7.34E-02	-4.79E-04	-1.09E-03	9.83E-03
InstruSupp	2.41E-02	7.87E-02	3.23E-03	5.64E-04	1.32E-02
NEOFAC_N	2.10E-02	6.76E-02	-8.30E-04	-1.37E-03	1.51E-02
FearAffect	1.81E-02	5.68E-02	-1.15E-03	-1.53E-03	1.06E-02
Friendship	1.79E-02	5.80E-02	-2.38E-03	-6.62E-04	1.50E-02
PercHostil	1.75E-02	6.79E-02	3.63E-03	7.90E-04	7.86E-03
PercStress	1.74E-02	7.21E-02	5.19E-05	-8.07E-04	9.97E-03
NEOFAC_A	1.66E-02	7.70E-02	3.80E-03	9.86E-04	1.30E-02
Sadness	1.38E-02	7.41E-02	2.84E-03	-1.61E-04	9.89E-03
ER40FEAR	1.22E-02	6.66E-02	4.57E-03	2.68E-04	1.08E-02
PosAffect	1.05E-02	7.08E-02	2.53E-03	9.00E-04	1.09E-03
raw_pain	9.80E-03	7.42E-02	3.95E-03	-1.25E-05	1.31E-03
NEOFAC_E	8.63E-03	8.25E-02	6.91E-03	3.28E-03	5.81E-03
NEOFAC_C	7.20E-03	7.91E-02	4.19E-03	1.50E-03	8.52E-04
FearSomat	4.72E-03	7.54E-02	2.45E-03	1.04E-03	-4.96E-03
ER40NOE	4.19E-03	8.17E-02	9.23E-04	-1.23E-03	-1.42E-03
PercReject	3.71E-03	7.29E-02	1.28E-03	1.51E-04	-8.64E-03
csq_psqi_se	2.78E-03	8.00E-02	2.21E-03	-4.72E-04	-6.20E-03
SelfEff	-3.43E-04	6.64E-02	1.75E-03	-1.77E-04	-1.12E-02
EmotSupp	-7.92E-04	7.36E-02	4.58E-03	1.36E-03	-5.55E-03
MeanPurp	-1.32E-03	7.30E-02	2.63E-03	5.55E-04	-4.03E-03

Table S15. Numerical improvement in prediction performance (COD) of multilayer meta-matching on each HCP-Aging phenotypes (K = 100). Rows were sorted by on the improvement of multilayer meta-matching over classical KRR (from largest to smallest). Negative values imply that multilayer meta-matching perform worse than baseline. The last column corresponds to the actual prediction accuracy of multilayer meta-matching.

References

Akiba, T., Sano, S., Yanase, T., Ohta, T., & Koyama, M. (2019). Optuna: A next-generation hyperparameter optimization framework. *Proceedings of the 25th ACM SIGKDD International Conference on Knowledge Discovery & Data Mining*, 2623–2631.
